# Deep learning-assisted high-content screening identifies isoliquiritigenin as an inhibitor of DNA double-strand breaks for preventing doxorubicin-induced cardiotoxicity

**DOI:** 10.1186/s13062-023-00412-7

**Published:** 2023-10-09

**Authors:** Xuechun Chen, Changtong Liu, Hong Zhao, Yigang Zhong, Yizhou Xu, Yi Wang

**Affiliations:** 1 Department of Cardiology, Affiliated Hangzhou First People’s Hospital, Zhejiang University School of Medicine, Hangzhou, 310006 China; 2 Pharmaceutical Informatics Institute, College of Pharmaceutical Sciences, Zhejiang University, Hangzhou, 310058 China; 3 College of Pharmaceutical Sciences, Zhejiang University, Hangzhou, 310058 China; 4 Innovation Institute for Artificial Intelligence in Medicine of Zhejiang University, Hangzhou, 310020 China; 5 Future Health Laboratory, Innovation Center of Yangtze River Delta, Zhejiang University, Jiaxing, 314100 China

**Keywords:** High-content screening, Isoliquiritigenin, Deep learning, Double-strand break, Anthracycline-induced cardiotoxicity

## Abstract

**Background:**

Anthracyclines including doxorubicin are essential components of many cancer chemotherapy regimens, but their cardiotoxicity severely limits their use. New strategies for treating anthracycline-induced cardiotoxicity (AIC) are still needed. Anthracycline-induced DNA double-strand break (DSB) is the major cause of its cardiotoxicity. However, DSB-based drug screening for AIC has not been performed possibly due to the limited throughput of common assays for detecting DSB. To discover new therapeutic candidates for AIC, here we established a method to rapidly visualize and accurately evaluate the intranuclear anthracycline-induced DSB, and performed a screening for DSB inhibitors.

**Results:**

First, we constructed a cardiomyocyte cell line stably expressing EGFP-53BP1, in which the formation of EGFP-53BP1 foci faithfully marked the doxorubicin-induced DSB, providing a faster and visible approach to detecting DSB. To quantify the DSB, we used a deep learning-based image analysis method, which showed the better ability to distinguish different cell populations undergoing different treatments of doxorubicin or reference compounds, compared with the traditional threshold-based method. Subsequently, we applied the deep learning-assisted high-content screening method to 315 compounds and found three compounds (kaempferol, kaempferide, and isoliquiritigenin) that exert cardioprotective effects in vitro. Among them, the protective effect of isoliquiritigenin is accompanied by the up-regulation of HO-1, down-regulation of peroxynitrite and topo II, and the alleviation of doxorubicin-induced DSB and apoptosis. The results of animal experiments also showed that isoliquiritigenin maintained the myocardial tissue structure and cardiac function in vivo. Moreover, isoliquiritigenin did not affect the killing of HeLa and MDA-MB-436 cancer cells by doxorubicin and thus has the potential to be a lead compound to exert cardioprotective effects without affecting the antitumor effect of doxorubicin.

**Conclusions:**

Our findings provided a new method for the drug discovery for AIC, which combines phenotypic screening with artificial intelligence. The results suggested that isoliquiritigenin as an inhibitor of DSB may be a promising drug candidate for AIC.

**Supplementary Information:**

The online version contains supplementary material available at 10.1186/s13062-023-00412-7.

## Background

The discovery of daunomycin, an antitumor anthracycline, in the 1950s [1] and its success in clinical trials for leukemia in the 1960s [2] were major breakthroughs in the field of oncology. Subsequently, other anthracycline compounds including doxorubicin (DOX) were discovered. DOX is the 14-hydroxy derivative of daunomycin and was identified as an even more potent antitumor agent [3]. To date, anthracyclines including DOX, daunomycin, idarubicin, and epirubicin are the key active components of many chemotherapy regimens for a variety of cancers, such as lymphoma, sarcoma, breast cancer, and childhood leukemia [4].

On the other hand, the cardiotoxicity of anthracyclines limits their use. Empirical data suggests that the cumulative dose of DOX should be limited to less than 450 ~ 550 mg/m^2^ [5]. However, cardiotoxicity was observed even after lower doses of DOX administration, with the early development of subclinical pathologic changes in endomyocardial biopsies [6], subclinical abnormalities of cardiac function [7], and chronic progressive cardiac contractile deficit years after DOX therapy [8].

To address the limitations of DOX, new approaches to treating anthracycline-induced cardiotoxicity (AIC) are needed. Dexrazoxane (DXZ), an FDA-approved orphan drug, may increase chemotherapy-induced myelosuppression while preventing AIC [9]. Another concern is that DXZ may diminish the chemotherapeutic efficacy of anthracyclines and increase the risk of second malignant neoplasms [10]. Thus, the medical need to develop new therapies and discover possible therapeutic cardioprotective agents remains unmet.

Although the exact pathomechanism of AIC remains unclear, several hypotheses have been proposed, including the production of reactive oxygen species (ROS) and the inhibition of topoisomerases [4]. The ROS hypothesis, also known as the iron hypothesis, is the traditional explanation for AIC. The quinone part of anthracyclines is catalyzed by NADH dehydrogenase into a reactive semiquinone radical, which is oxidized to generate superoxide anions (O_2_^·−^) and hydrogen peroxide (H_2_O_2_). In the presence of iron, hydrogen peroxide (H_2_O_2_) produces highly toxic hydroxyl radicals (HO^•^) via the Fenton reaction [11]. Peroxynitrite (ONOO^−^) was generated by the reaction of superoxide anions (O_2_^·−^) with nitric oxide (NO), which is also an important culprit for DNA strand breaks [12]. ROS and ONOO^−^ both cause oxidative damage to biological macromolecules including DNA, ultimately leading to cardiomyocyte cellular dysfunction and cell death. Recently, the topoisomerase hypothesis has been proposed as a new mechanism of AIC. Anthracyclines bind to DNA and topoisomerase II (topo II), which hinders the re-ligation of the DNA double strands, and persistent DNA double-strand break (DSB) was generated consequently. DSB is lethal to cells and brings about p53 activation and cell death [13]. Zhang et al. [14] found that the deletion of topo IIβ from cardiomyocytes prevented mice from developing AIC, suggesting the critical role of topoisomerase-mediated DSB in the pathomechanism of AIC.

DSB is a pathological link involved in both the ROS hypothesis and the topoisomerase hypothesis. Persistent DSB leads to the activation of apoptotic pathways and the loss of cardiomyocytes in the heart [15], which initially manifests as subclinical myocardial damage that gradually leads to an early asymptomatic reduction in the left ventricular ejection fraction and eventually to symptomatic, often refractory heart failure [16]. Therefore, anthracycline-induced DSB represents one of the important mechanisms of AIC, which provides a new promising direction for AIC prevention and treatment [15].

However, DSB measurement-based drug discovery for cardioprotectors against AIC has not been reported as of 2023. While previous screening methods based on cell viability have effectively identified compounds that improve the ultimate fate of the cardiomyocytes, they could not provide enlightenment on the mode of action of the candidate compounds. The lacking of DSB-based AIC drug screenings is possibly due to the difficulty in the high-throughput detection of DSB. The comet assay can accurately and directly detect DSB in cells, but it is labor-intensive [17] and thus can hardly meet the needs of modern high-throughput drug screening. With a deeper understanding of the molecular mechanism of DSB and the advancement of high-content imaging technology, it has become possible to quantify DSB by high-throughput imaging after fluorescent labeling of the molecular markers of DSB. For example, phosphorylated histone H2AX (γ-H2AX) foci and p53-binding protein 1 (53BP1) foci are accepted markers of DSB [18, 19]. Among them, 53BP1 foci can be observed not only by antibody-based immunofluorescence, but also by fluorescent protein-based methods. By overexpressing fluorescent protein along with the region of 53BP1 required for its recruitment, the DSB sites in cells can be visualized. Fluorescent protein-based method does not need the tedious steps that are required for immunofluorescence, and is easier to extend to high-throughput assays.

In this study, we describe a new assay platform for the high-throughput screening of reagents that modulate the level of DSB in DOX-damaged cardiomyocytes. One of the challenges here is the difficulty of analyzing foci images. In our previous work, we used deep learning to identify irradiation-induced DNA damage in the HeLa cell line [20]. We extend our work to DOX-induced DNA damage in cardiomyocytes. In this study, twelve active compounds that reduce 53BP1 foci were identified from 315 compounds, three of which were proved to be cardioprotective. We then verified that isoliquiritigenin (ISL) possesses cardiomyocyte protective activity, reduces DSB levels, and alleviates DOX-induced damage in both cardiomyocytes and mouse hearts. Furthermore, we found that the anti-DSB effect of ISL is potentially correlated with its activation of antioxidative heme oxygenase-1 (HO-1), the inhibition of topo II, and the inhibition of Bcl-2 associated X-protein (BAX) and Caspase 3. Overall, the artificial intelligence-assisted high-content screening method to evaluate DNA damage in doxorubicin-induced cardiotoxicity was established for the first time. Using the established method, we discovered isoliquiritigenin, which could be a potential lead compound against anthracycline-induced cardiotoxicity.

## Results

### High-content imaging detected doxorubicin-induced DNA double-strand breaks in H9c2 cardiomyocytes

**Fig. 1:**
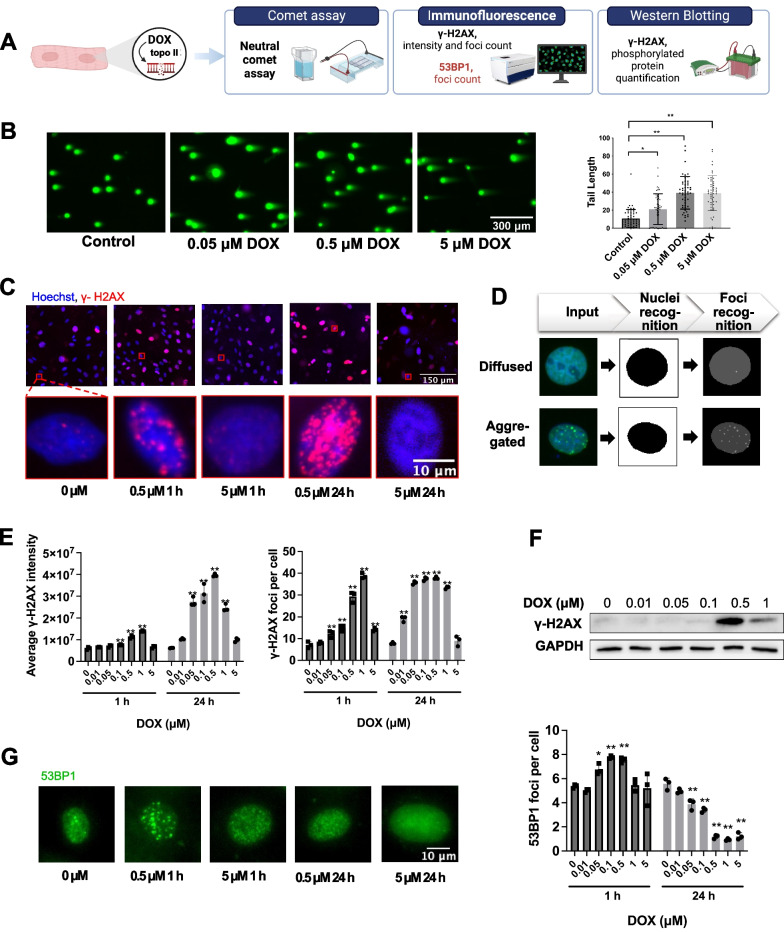
High-content imaging can detect doxorubicin-induced DNA double-strand breaks in H9c2 cardiomyocytes. **A** DOX-induced DSB was detected by different methods. **B** Comet assay results of H9c2 after incubation with DOX for 24 h. Data are plotted as mean ± SD, n = 50 (50 cells). Kruskal–Wallis test was performed, *P < 0.05, **P < 0.01. **C** Representative images of H9c2 after incubation with 0.5 μM or 5 μM DOX for 1 h or 24 h, γ-H2AX labeled by immunofluorescence. Representative images of cells treated with other different concentrations of DOX were shown in Additional file 1: Fig. S1A. **D** Intranuclear foci were recognized by the threshold-based traditional image analysis method. **E** Analysis results of γ-H2AX immunofluorescence images. The number of cells that were captured and quantified was shown in Additional file 1: Fig. S1B.** F** H9c2 was incubated with DOX for 24 h and the γ-H2AX (Ser139) phosphorylated protein (15 kDa) was detected and GAPDH (36 kDa) was used as the loading control. The quantified results of three experiments were shown in Additional file 2: Fig. S2A. **G** H9c2 was incubated with different concentrations of DOX for 1 h or 24 h, followed by the immunofluorescence labeling and analysis of 53BP1. The number of cells that were captured and quantified was shown in Additional file 1: Fig. S1C. For **E** and **G**, data are plotted as mean ± SD, n = 3, *P < 0.05, **P < 0.01 when compared with the vehicle (dimethyl sulfoxide, DMSO)-treated control group

To determine the feasibility of 53BP1 labeling and high-content imaging in detecting DOX-induced DSB, we compared 53BP1 imaging with three other commonly used DSB detection methods: neutral comet assay, γ-H2AX immunofluorescence assay, and relative quantification of γ-H2AX protein (Fig. 1A).

The neutral comet assay represents the direct detection of DSB in cells using gel electrophoresis [21]. The results of the neutral comet assay showed that the comet tail length increased with increasing DOX concentration after 24 h of treatment, representing an increase in the degree of DSB (Fig. 1B).

DNA damage response occurs after DSB and is marked by increased levels of H2AX phosphorylation [22] and more γ-H2AX/53BP1 foci [23]. γ-H2AX is synthesized de novo after DSB happened, whereas 53BP1 is present all the time, diffusely distributed in the nucleus in normal cells, and aggregates at DSB sites to form foci after DSB [24]. We performed immunofluorescence on γ-H2AX (Fig. 1C, Additional file 1: Fig. S1A) and used the traditional threshold-based method to analyze the number of foci (Fig. 1D). The immunofluorescence images and quantification results showed that γ-H2AX, both in its protein level (Fig. 1E left) and foci number (Fig. 1E right), varied with the concentration and duration of DOX treatment, which was consistent with the trend of γ-H2AX protein level demonstrated by western blotting (Fig. 1F). At a high dose of DOX, the γ-H2AX intensity decreased, which is in accordance with the literature [25, 26]. The decrease of phosphorylated γ-H2AX by high-dose DOX may be due to the fact that the decreased activity of proteasomes causes the DSB sites to fail to expose from topo IIβ, such that the DNA damage response cannot be fully triggered [27].

Similar to γ-H2AX, the 53BP1 foci number also varied with the dose and duration of DOX treatment (Fig. 1G). What is more, the change of foci number could reflect low-level DSB in cells treated with DOX as low as 0.05 μM for 1 h, which suggested that the foci assay represents a more sensitive assay than the comet assay. Therefore, we suggest that DOX-induced 53BP1 foci aggregation can be developed as a high-throughput and sensitive assay to quantify DNA damage in cells on a large scale, by which we could discover inhibitors of DOX-induced DSB with cardioprotective potential.

### A high-content imaging method for doxorubicin-induced DNA damage was developed based on fluorescent protein labeling

**Fig. 2:**
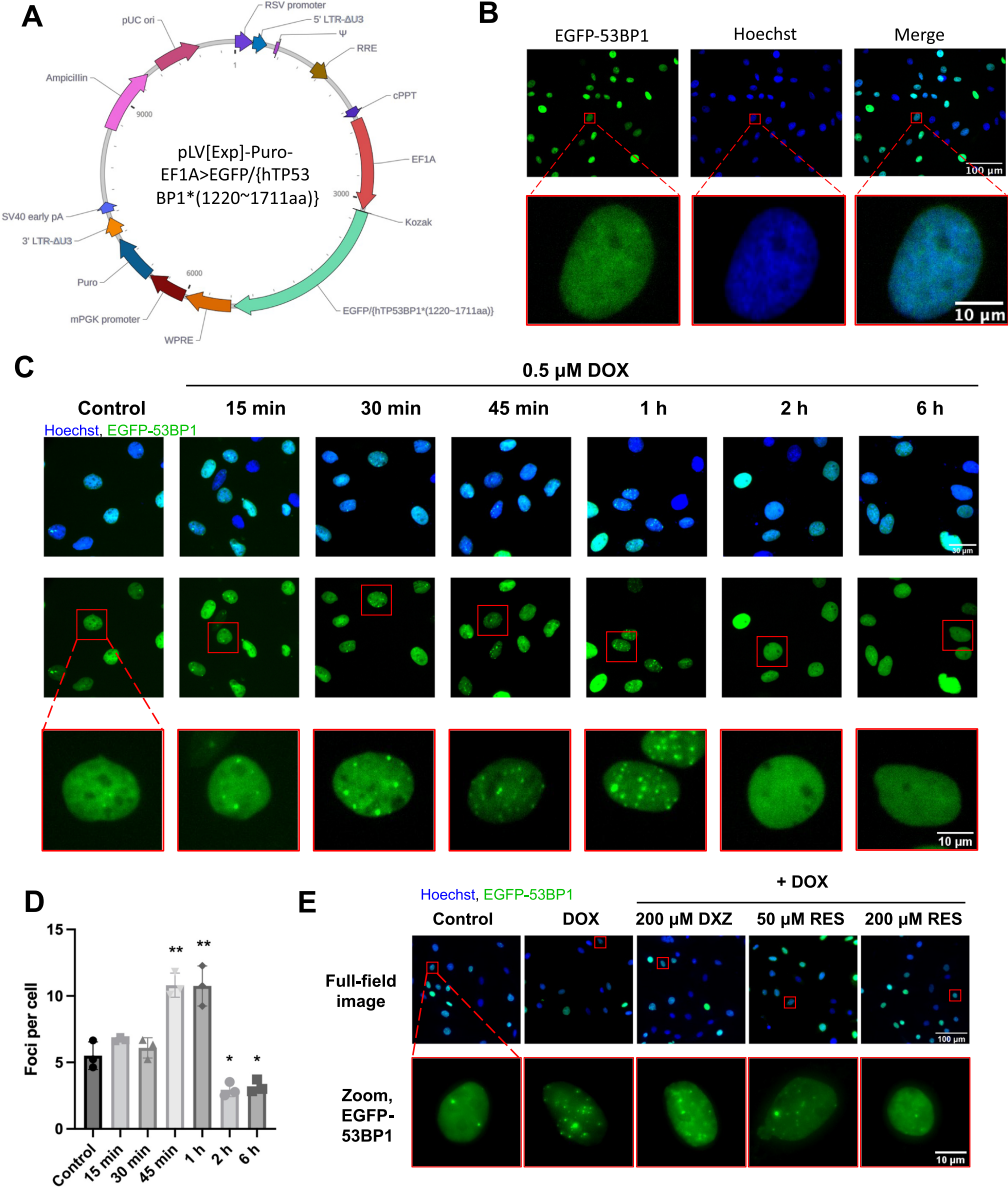
Construction of a high-content imaging method for doxorubicin-induced DNA damage by labeling with fluorescent protein. **A** Map of the lentiviral vector for a stable expression of EGFP-53BP1 (1220 ~ 1711 aa).** B** Representative two-color fluorescent images of EGFP-53BP1-H9c2. **C** Distribution of 53BP1 after the treatment of EGFP-53BP1-H9c2 with 0.5 μM DOX for different time durations. **D** The experimental images in (C) were analyzed by the traditional method. Data are plotted as mean ± SD, n = 3, *P < 0.05, **P < 0.01 when compared with the control group. **E** Effect of dexrazoxane (DXZ) or resveratrol (RES) on the distribution of 53BP1 in EGFP-53BP1-H9c2 treated with 0.5 μM DOX for 1 h

To efficiently label DNA damage foci in the nucleus and avoid the complex staining process and batch-to-batch variation introduced by antibodies, we constructed an H9c2 cardiomyocyte cell line stably expressing EGFP-53BP1 (1220 ~ 1711 aa), which constitutively expresses the fusion protein of the enhanced green fluorescent protein (EGFP) and the minimal focus-forming region (1220 ~ 1711 aa) of 53BP1 [28] so that the distribution of EGFP gives an indication of where 53BP1 is recruited. This cell line was obtained using lentiviral transduction, and the vector map is shown in Fig. 2A. After obtaining EGFP-53BP1-H9c2 cells, we labeled the nucleus with Hoechst, and the green fluorescent EGFP signal was located in the nucleus (Fig. 2B), consistent with the fact that 53BP1 is localized in the nucleus.

Since the clinical blood concentration of DOX ranged from 0.1 to 1 μM [29, 30], we chose 0.5 μM as the concentration of DOX in the model group. When the cells were treated with 0.5 μM DOX for 1 h, significant 53BP1 aggregation could be seen in the nucleus (Fig. 2C), consistent with the immunofluorescence result by use of the anti-53BP1 antibody in Fig. 1G. This indicates that the method of foci imaging with the constructed H9c2-EGFP-53BP1 cell line was also able to recapitulate DOX-induced DSB like the immunofluorescence assay. We calculated the average number of foci per cell, which was significantly increased in the 1 h DOX-treated group compared with the control group (Fig. 2D).

To further demonstrate the reliability of the model, DXZ and resveratrol (RES) were selected as reference compounds, of which DXZ is the FDA-approved drug for the prevention of DOX cardiomyopathy, while RES is an active compound that has been reported as an anti-AIC candidate [31], both of them were reported to have anti-doxorubicin effect in H9c2 [32]. Compared with the model group (0.5 μM DOX treatment for 1 h), 53BP1 aggregation was alleviated in the DOX + reference compound group (Fig. 2E), indicating that the cell model is able to reliably reflect the protective effect of the active compounds.

### Deep learning-based method recognized the DNA damage status at single-cell level and correctly reflected the protection effect of reference compounds

The current traditional methods for the recognition of nuclei boundaries and the recognition of foci are mostly threshold-based [33]. In term of recognizing nuclei boundaries, when different nuclei have different Hoechst staining brightness, it is hard to accurately identify the boundaries of all nuclei by the same threshold (Additional file 3: Fig. S3). In term of recognizing EGFP-53BP1 foci, the green fluorescence differs in brightness among different EGFP-53BP1-H9c2 cells, and is even not detected in some cells, which makes the threshold-based method no longer capable (Additional file 4: Fig. S4).

**Fig. 3:**
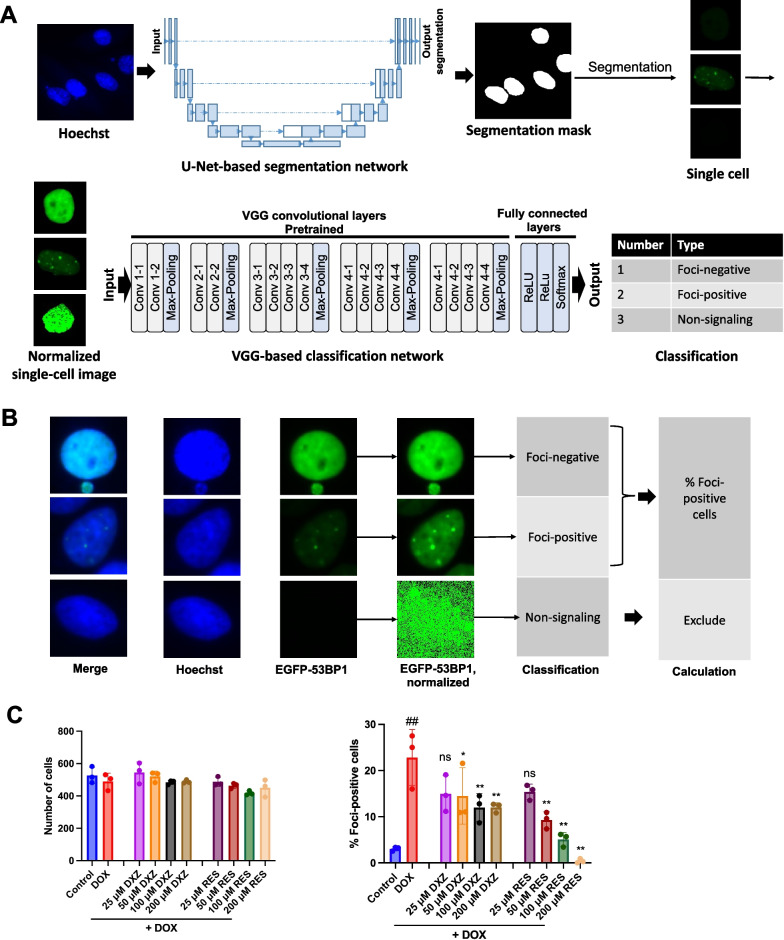
Deep learning to evaluate the DNA damage of single cells and the effect of reference compounds. **A** Schematic of the deep learning-based image analysis method. **B** Representative images of single cells of the three categories.** C** Quantitative results of the effect of reference compounds on foci generation using the above deep learning-based image analysis method. Data are plotted as mean ± SD, n = 3, ^##^*P* < 0.01 when compared with the control group, and **P < 0.01 when compared with the DOX group

We compared the threshold-based traditional method with the deep learning-based method, FociNet, that we developed earlier. The quantitative methodologies are as follows. In the threshold-based traditional method, the Granularity module in MetaXpress software was used to recognize nuclei and foci, and then the number of foci per cell was calculated. In the deep learning-based method (FociNet), to obtain more accurate discrimination of cellular DNA damage status, we first segmented the images into single-cell images by U-Net-based convolutional neural network and then classified the normalized single-cell images into different categories by the VGG-19-based network. Individual cells with different brightness were normalized and classified into three categories (foci-positive, foci-negative, and non-signaling cells) based on their EGFP distribution patterns (Fig. 3A), and this was not disturbed by the difference in brightness between cells (Fig. 3B). The classification of each single cell was obtained by running FociNet, and the percentage of foci-positive cells (% foci-positive cells) was calculated.

We used the two different methods to analyze the image set corresponding to Fig. 2E. This image set includes images from three replicated experiments, with hundreds of cells captured per experiment. The quantitative results were shown in Additional file 5: Fig. S5A and Fig. 3C. It can be seen from the results that when dealing with the image set corresponding to Fig. 2E, the ability of the threshold-based method to distinguish differences between different groups is not satisfactory (Additional file 5: Fig. S5A). The deep learning-based method, when applied to the analysis of corresponding images, can effectively distinguish the cell populations of the reference compound group from that of the DOX group and reflect the dose-dependent protective effect of the reference compounds (Fig. 3C).

### High-content screening identified protective agents against doxorubicin-induced DNA damage

**Fig. 4:**
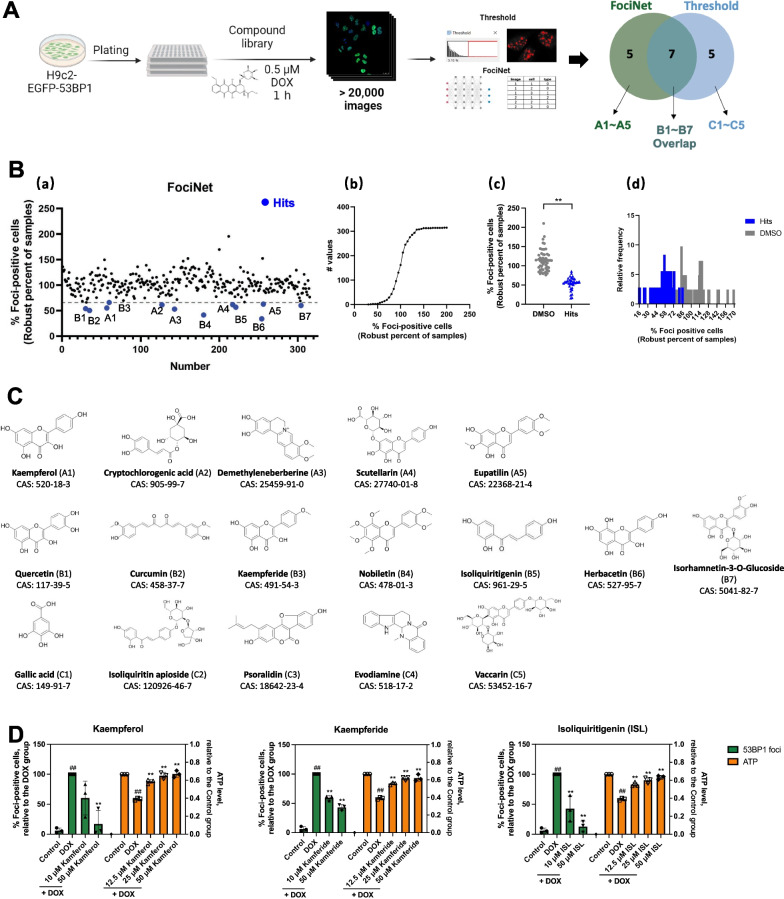
Deep learning-based high-content screening to find protective agents against doxorubicin-induced DNA damage.** A** Schematic of the high-content screening. **B **(a) Scatter plot of the primary screening results, where the mean values of the % foci-positive cells analyzed by FociNet are presented as robust percent of samples (RPS), and the blue data points are hit compounds. (b) Cumulative distribution of the primary screening data analyzed by the FociNet. (c) DMSO-treated control wells vs. hit compounds-treated wells. Data are plotted as mean ± SD, n = 36 ~ 54, **P < 0.01 by Mann–Whitney test. (d) Frequency distribution of data from DMSO-treated control wells vs. hit compounds-treated wells. **C** Seventeen hit compounds from the primary screening. **D** Effect of kaempferol, kaempferide, and ISL on foci formation induced by 0.5 μM DOX treatment for 1 h in EGFP-53BP1-H9c2, and that on ATP decrease induced by 0.5 μM DOX treatment for 24 h in H9c2. Data are plotted as mean ± SD, n = 3, ^##^*P* < 0.01 when compared with the control group; **P < 0.01 when compared with the DOX group

Using the constructed high-content imaging method and image analysis method, we screened a library containing 315 compounds, which had been prepared by our lab previously. For the primary screening, each compound was co-incubated at a dose of 50 μM with 0.5 μM DOX for EGFP-53BP1-H9c2 for 1 h and tested 3 times. We obtained more than 20,000 two-color images, then used a threshold-based traditional method to analyze and calculate foci per cell, and also used the deep learning-based method (FociNet) to analyze and calculate the percentage of foci-positive cells. The primary screening yielded 17 hit compounds that reduced foci, with 7 hit compounds overlapping between the two image analysis methods (Fig. 4A).

As expected, the screening data were normally distributed, and the number of foci of the hit compounds is lower than that of the DMSO-treated controls (Fig. 4B and Additional file 5: Fig. S5B,C). The molecular formulae, names, and CAS numbers of the 17 primary hit compounds were shown in Fig. 4C. In the secondary screening, most of the 17 primary hit compounds were verified to reduce foci and 12 of them showed a significant foci-reducing effect at the concentration of 50 μM (Additional file 6: Fig. S6). To clarify whether the hit compounds had cardioprotective effects, we tested the ATP levels of H9c2 treated with the 12 compounds. Three of the 12, namely kaempferol, kaempferide, and isoliquiritigenin (ISL), not only reduced the foci but also ameliorated the DOX-induced decrease in ATP levels in H9c2 cells in a dose-dependent manner (Fig. 4D), thus exhibiting cardioprotective potential. Since ISL was first found to be associated with preventing DOX-induced DNA damage, we then investigated it further.

### ISL attenuated doxorubicin-induced DNA damage

**Fig. 5:**
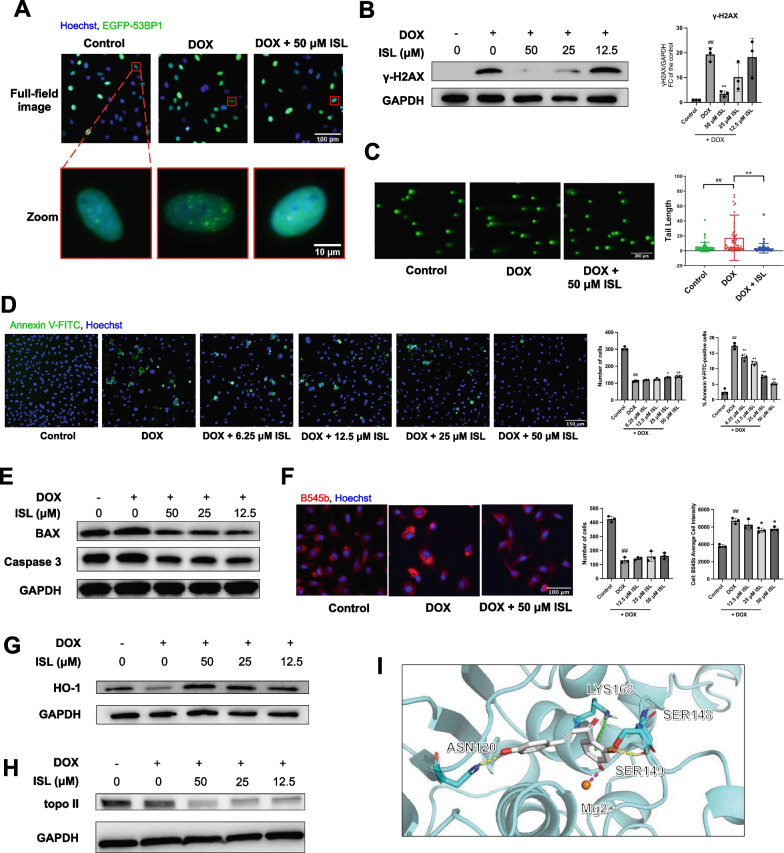
ISL inhibits DNA damage and attenuates doxorubicin-induced cardiotoxicity in H9c2 cardiomyocytes.** A** Dose-dependent influence of ISL on foci formation in EGFP-53BP1-H9c2 treated with 0.5 μM DOX for 1 h. **B** Effect of ISL on the level of phosphorylated γ-H2AX (Ser139) protein (15 kDa). **C** Effect of ISL on the level of DSB detected by the comet assay. **D** Effect of ISL on the proportion of apoptotic/necrotic cells, which were labeled with annexin V-FITC. **E** Effect of ISL on the protein levels of BAX (21 kDa) and Caspase 3 (35 kDa). **F** Effect of ISL on the cellular peroxynitrite labeled with B545b. **G** Effect of ISL on the level of HO-1 protein (28 kDa). **H** Effect of ISL on the level of topo II protein (180 kDa). The lower band with lower molecular weight was topo IIα, and the upper band with larger molecular weight was topo IIβ. For (**B**) ~ (**G**), H9c2 was incubated with 0.5 μM DOX and certain concentrations of ISL for 24 h. For (**H**), H9c2 was incubated with 0.5 μM DOX and certain concentrations of ISL for 1 h. For (**B**), (**D**), and (**F**), data are plotted as mean ± SD, n = 3, and data were analyzed by ANOVA. For (C), data are plotted as mean ± SD, n = 62, and data were analyzed by Kruskal–Wallis test. ^#^*P* < 0.05, ^##^*P* < 0.01 when compared with the control group; *P < 0.05, **P < 0.01 when compared with the DOX group. **I** Molecular docking results of ISL to topo II. Salt bridge: purple line; hydrogen bond: yellow line; cation-π: green line. The quantified results of western blotting in (**E**), (**G**) and (**H**) were shown in Additional file 2: Fig. S2B–E

To confirm that ISL reduces DNA damage, we verified its dose-dependent reduction of 53BP1 foci in foci imaging (IC_50_ = 43.65 μM, see Fig. 5A and Additional file 7: Fig. S7A), as well as its reduction of H2AX phosphorylation in western blotting (Fig. 5B) and its reduction of DSB in neutral comet assay (Fig. 5C), which confirmed its anti-DNA damage effect.

Subsequently, we found that ISL significantly reduced the percentage of Annexin V-FITC-positive apoptotic/necrotic cells under DOX stimulation (IC_50_ = 21.81 μM, see Fig. 5D and Additional file 7: Fig. S7B), which is associated with the reduced protein levels of the pro-apoptosis-related proteins BAX and Caspase 3 (Fig. 5E).

Peroxynitrite is a potent oxidant that can cause DNA strand breaks and is reported as one of the major triggers of DOX-induced cell death [34]. DOX markedly increased the amount of peroxynitrite inside H9c2, which was reduced by the treatment of ISL (Fig. 5F). We also determined the effects of ISL on HO-1 and observed that the HO-1 protein level was increased in the ISL-treated group (Fig. 5G).

Topo II is a key mediator in the pathological process of the production of DOX-induced DSB. Previous studies have shown that either genetic ablation [14] or pharmacological reduction [35] of topo II mediates myocardial tolerance to DOX. We found that the application of 50 μM ISL for 1 h significantly reduced the protein levels of topo II (α and β) (Fig. 5H). Next, we simulated the interaction of ISL with topo II. The molecular docking results are shown in Fig. 5I. There are four amino acid residues around the ligand, ISL. Among them, ASN120, SER148, and SER149 formed hydrogen bonds with the ligand, LYS168 formed cation-π with the benzene ring of the ligand, and the magnesium ion formed salt bridges with the ligand, with a docking score of -9.534, indicating a predicted strong binding of ISL to topo II.

**Fig. 6:**
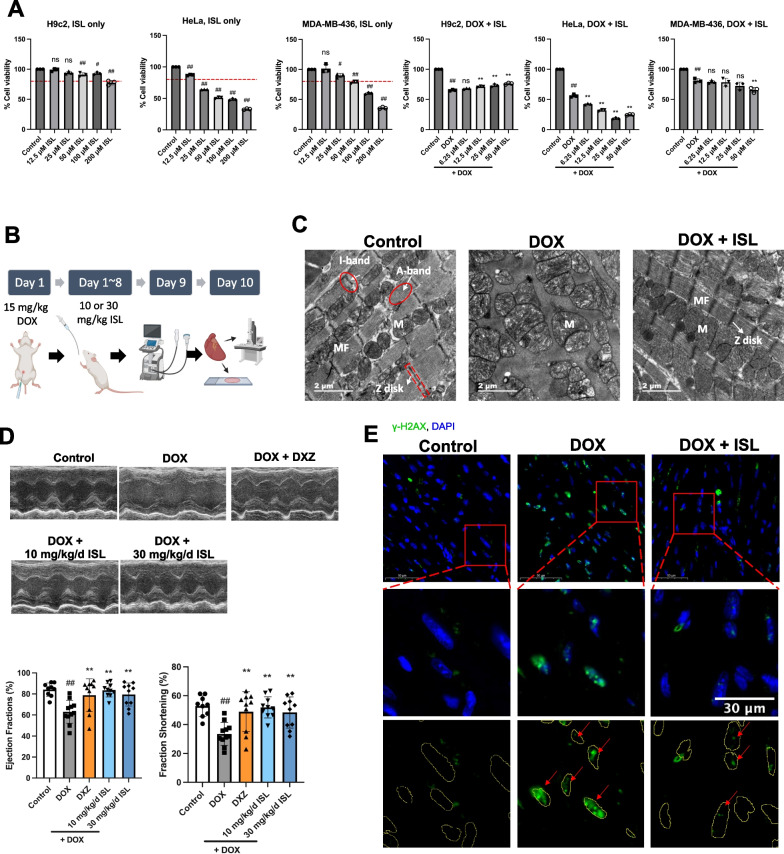
ISL exerted different effects on cardiomyocytes and cancer cells, and protected mice from doxorubicin-induced cardiotoxicity.** A** ISL exhibits different effects on H9c2 cardiomyocytes and cancer cells (HeLa and MDA-MB-436). The cell viability was detected by MTT assay after the incubation of cells with different concentrations of ISL with or without 0.5 μM DOX for 24 h. The red dotted line indicates 80% cell viability. Data are plotted as mean ± SD, n = 3, ^#^*P* < 0.05, ^##^*P* < 0.01 when compared with the control group, and **P < 0.01 when compared with the DOX group. **B** Schematic of the animal experiment. **C** Representative transmission electron microscopy images of myocardial tissue from control, DOX-treated, and DOX + ISL-treated mice. **D** Representative echocardiographic images from different groups of mice, with the quantitative results below. Data are plotted as mean ± SD, n = 9 to 11, ^##^*P* < 0.01 when compared with the control group; **P < 0.01 when compared with the DOX group. **E** γ-H2AX was labeled by immunofluorescence staining on paraffin-embedded heart tissue sections

To determine whether ISL affects the antitumor effect of DOX, we further tested the effect of ISL on the viability of HeLa cervical cancer cells and MDA-MB-436 breast cancer cells. Compared with H9c2, ISL reduced HeLa cell viability to below 80% at a much lower concentration, indicating a potential antitumor effect. When administered along with DOX, ISL alleviated the viability reduction by DOX in H9c2, but reduced HeLa viability to an even lower level, suggesting that ISL did not affect the antitumor effect of DOX on HeLa (Fig. 6A). The effect of ISL on MDA-MB-436 was similar to that on HeLa, which further illustrates the potential of ISL administration along with DOX chemotherapy.

We then tested the efficacy of ISL on DOX-induced acute myocardial injury in mice by the intraperitoneal injection of DOX (15 mg /kg) followed by intragastric administration of ISL for 8 days (Fig. 6B). The ultrastructure of myocardial myofilaments and mitochondria was observed by transmission electron microscopy (Fig. 6C). In the myocardium of mice in the control group, the ultrastructure was normal and showed the following phenomena: myofilaments (MF) were neatly arranged in the myocardial cells, and mitochondria (M) were orderly arranged on both sides of the myofilaments. The bright I band of myofilaments alternated with the dark A band, and a distinct Z disk could be observed in the center of the bright band. In the myocardium of mice in the DOX group, the ultrastructure of myofilaments was damaged and the mitochondria were swollen. Compared with the DOX group, the myofilament structure was more intact and the swelling of mitochondria was reduced in the DOX + ISL group. Echocardiographic evidence confirmed the decreased cardiac function in the mice of the DOX group. The ejection fractions and fractional shortening were significantly lower in the DOX group compared with the normal control group. Compared with the DOX group, the DOX + ISL group showed higher ejection fractions and fractional shortening (Fig. 6D). In the myocardial tissue of the mice in the DOX group, there was remarkable intranuclear H2AX phosphorylation and γ-H2AX foci, suggesting the presence of DSB. In the DOX + ISL group, there was less visible intranuclear γ-H2AX foci, suggesting an attenuated DSB (Fig. 6E) and providing further insights for DNA damage at the animal level. In conclusion, the results demonstrate the cardioprotective effect of ISL in vivo.

## Discussion

DSB mediated by topo II in cardiac myocytes is a key pathological process in AIC. However, to date, high-throughput screening methods to evaluate the effects of agents on DOX-induced DSB have not been developed. Herein, we provide a high-throughput, high-content assay platform that presents an effective way to discover novel therapies to mitigate DOX cardiotoxicity.

Both γ-H2AX foci and 53BP1 foci are widely accepted markers for DSB. We focus on the 53BP1 foci instead of γ-H2AX foci because the 53BP1 foci can be detected by overexpressing fluorescent fusion proteins. The fluorescent protein-based approach has a higher resistance to photobleaching, and avoids the steps required for immunofluorescence such as permeabilization, blocking, antibody incubation, etc., so it has a higher speed, a lower cost, a lower operation error, thus it is easier to be expanded to high-throughput assays.

We successfully obtained H9c2 with stable expression of the fluorescent fusion protein (EGFP-53BP1) by lentiviral transfection. Most of the cells still expressed the EGFP-53BP1 fusion protein that is visible under fluorescence microscopy after many passages. So, a large number of cells for high-content screening can be produced by cell passaging and proliferating. Nevertheless, the green fluorescence intensity varied among different cells, and in some cells even no green fluorescence was detected. The cell-to-cell variance in the intensity of fluorescent protein is also commonly reported in other cells stably transfected with fluorescent proteins [36]. The possible reasons include: (i) the copy number of the exogenous gene introduced into different cells is different; (ii) the activity of the promoters of the exogenous gene in different cells is different [37]. Besides, no green fluorescence was detected in some *EGFP-53BP1* transfected cells, even in the presence of puromycin. The possible reasons include: (i) EGFP-53BP1 expression could be too low to be detected at the set exposure time. In order to avoid overexposure, the exposure time is set according to most cells with bright green fluorescence, and at such an exposure time, the signal of cells with quite low EGFP-53BP1 expression would not be distinguishable from the background noise; (ii) Cellular epigenetic modification could lead to transcriptional silencing of *EGFP-53BP1.* According to the general principles of plasmid construction, in order to prevent interference with the expression efficiency and biological function of the target protein, the antibiotic resistance gene (in this case, the puromycin resistance gene) and the target gene (in this case, *EGFP-53BP1*) are generally located after two different promoters. Cells may block the binding of transcription factors to the promoters of the target gene through epigenetic modifications, resulting in transcriptional silencing of the target gene [38].

As a result of different expression of fluorescent protein between different cells, the difficulty of image analysis was increased. Even so, we have proved that the deep learning-based approach can solve the difficulty of image analysis of cells overexpressing fluorescent protein, and take full advantage of the high throughput of labeling with fluorescent protein. For instance, our detection and analysis pipeline has the following advantages: (i) higher throughput than the comet assay by the high-speed automatic image capturing of multiple microplates; (ii) the cells stably express fluorescent fusion proteins, providing a faster and lower-cost way to label DSB, compared with immunofluorescence; (iii) image analysis can be adapted to cells with different fluorescence brightness or even across experimental batches, eliminating the need to change the brightness threshold for different cells/experiments as compared with the traditional method; (iv) the image analysis can distinguish cells that do not express fusion proteins and prevent them from being incorrectly identified as 53BP1 diffusely distributed normal cells.

Despite the above advantages, the proposed platform has certain limitations. First, foci formation will be hindered by high-dose DOX, probably due to the activity decrease of proteasomes that impedes topo II degradation and DSB exposure, hence foci imaging is not applicable to DSB quantification at high DOX concentrations or long-term DOX treatment. Second, in the presence of reagents that interfere with the DNA damage response, the extent of 53BP1 aggregation may no longer represent the level of DSB, so a comet assay is needed to detect the actual level of DSB. However, we demonstrated that the screening platform can identify active DSB inhibitors under the specified conditions of DOX treatment.

Using the constructed platform, we screened a compound library to identify new anti-DOX cardioprotective compounds. Three compounds, kaempferol, kaempferide, and ISL, not only reduced 53BP1 aggregation in the screening assays, but were also validated to alleviate DOX-induced decrease in the cell viability of cardiomyocytes. Among them, ISL was verified to reduce the DSB and apoptosis of cardiomyocytes and alleviate cardiac injury in mice.

ISL (2′,4,4′-trihydroxychalcone) is a small molecule discovered from the traditional Chinese medicine Gan Cao (licorice, RADIX ET RHIZOMA GLYCYRRHIZAE). ISL has been found to possess various biological activities such as anticancer, antidiabetic, anti-inflammatory, and anti-influenza, with most studies related to its anticancer activity [39]. ISL has been previously reported to prevent DOX-induced liver injury and kidney injury in rats [40, 41]; however, it has not been reported to prevent DOX-induced DNA damage in cardiomyocytes. Our finding that ISL did not interfere with the DOX-induced decline of the viability of HeLa and MDA-MB-436 cancer cells highlights the possibility that the antitumor and cardiotoxic effects of DOX can be pharmacologically separated.

Peroxynitrite is one of the culprits of both DNA single-strand and double-strand breaks [42]. Superoxide anions (O_2_^·−^) produced by anthracyclines rapidly react with nitric oxide (NO) to yield peroxynitrite (ONOO^−^), which is a potent oxidant that reacts with proteins, lipids, and DNA. We observed that ISL significantly alleviated DOX-induced peroxynitrite production, which may partially explain its reduction of DSB. HO-1 has been reported to be the rate-limiting enzyme in heme degradation and can be increased by a variety of stressors, exerting a pre-adaptive effect; evidence has been presented that increased expression of HO-1 prevents the adverse cytotoxic effects caused by peroxynitrite [43]. The upregulation of HO-1 protein by ISL may explain the inhibitory effect of ISL on peroxynitrite production. It is worth noting that we observed that ISL reduced topo II protein level, which was also potentially associated with ISL’s ability to inhibit DSB. In this paper, we found that similar to DXZ [35], one hour incubation of ISL decreased topo II. Previously, researchers demonstrated that ISL was a catalytic inhibitor of topo II through in vitro topo II inhibition assay [44]. From the molecular docking results, we speculated the binding of ISL to topo II, and whether the decrease in topo II is due to ISL’s blocking effect of the topo II enzymatic reaction cycle remains to be discovered by further studies.

The ability to translate cellular and animal studies to the clinical treatment of patients with late-onset chronic AIC remains to be examined. More preclinical research, including efficacy evaluations of ISL on an animal model of DOX-induced delayed chronic cardiomyopathy, long-term toxicity studies and pharmacokinetic research, is needed before the successful clinical use of ISL for cardioprotection. In addition, to confirm that ISL does not affect the antitumor effects of DOX, testing on multiple tumor cells and multiple animal tumor models is needed.

## Conclusions

In conclusion, our screening method could identify active compounds that inhibit DNA DSB induced by DOX. In addition, we validated the anti-DSB effect and cardioprotective effect of ISL, thus providing a valuable screening method and source of lead compounds for new treatment options for DOX-induced cardiotoxicity.

## Materials and methods

### Cell culture and transfection

Rat cardiomyocyte cell line H9c2 (2–1) (H9c2 for short) was purchased from the Shanghai Cell Bank. Cervical cancer cell line HeLa and breast cancer cell line MDA-MB-436 were from ATCC (USA). DMEM complete medium was prepared by mixing DMEM medium (CORNING, New York, USA), fetal bovine serum (10% v/v, CORNING), and 100 × penicillin–streptomycin solution (1% v/v, CORNING). H9c2, HeLa and MDA-MB-436 were cultured in DMEM complete medium in a CO_2_ incubator at 5% CO_2_ and 37 °C. All cell lines were used within 10 passages after thawing from stocks.

The gene expression lentiviral vector of EGFP-53BP1 (1220 ~ 1711 aa) was constructed by directly linking EGFP to the gene sequence of human TP53BP1, and the vector map is shown in Fig. 2A. HEK 293 T cells were transfected to package the virus, and lentiviral particles were generated and collected. The construction of the gene expression lentiviral vector and the collection of lentiviral particles were performed by Yunzhou Biological Company (Guangzhou, China). Then, H9c2 was transfected to obtain H9c2 stably expressing EGFP-53BP1 (1220 ~ 1711 aa), which was called H9c2-EGFP-53BP1 and was cultured in a CO_2_ incubator at 5% CO_2_ and 37 °C using DMEM complete medium supplemented with 0.5 μg/mL puromycin (YEASEN, Shanghai, China). All cells were maintained mycoplasma-free.

### Comet assay

Neutral comet assays were performed to determine the level of DSB. First, a 0.8% normal melting-point agarose (YEASON) layer was prepared on frosted slides. Second, 30 μL of cell suspension (1.5 × 10^6^ cells/mL) was mixed with 80 μL of 1.5% low melting-point agarose (AMRESCO) and the mix was layered onto the precoated slides to form a cell-embedded agarose layer. The lysis was performed at 4 °C for 2 h. The lysed slides were subsequently soaked in TBE buffer for 30 min, and then electrophoresed in TBE for 20 min at 25 V, 15 mA. Finally, the slides were stained with Gel-Green (Beyotime, Shanghai, China), and images of more than 50 cells were acquired with a Leica DMi3000 B fluorescence microscope and analyzed by the OpenComet [45] plugin in ImageJ (National Institutes of Health).

### Immunofluorescence assay

H9c2 cells were seeded in 96-well plates with 2000 cells or 3000 cells per well. After certain treatments, cells were fixed with 4% paraformaldehyde solution (Sangon, Shanghai, China) for 20 min, permeabilized with 0.2% Triton X-100 for 10 min, blocked with PBST (PBS + 0.1% Tween-20) containing 1% BSA and 22.52 mg/mL glycine for 30 min, then incubated with primary antibody at 4 °C overnight. Subsequently, cells were incubated with fluorophore-coupled secondary antibody at room temperature for 2 h, and finally with 1 μg/mL of Hoechst 33,342 (Invitrogen, Carlsbad, USA) for 10 min to stain the nuclei. Images were acquired by the ImageXpress Micro Confocal (Molecular Devices, San Jose, USA) high-content imaging system using a 40× objective. Images were analyzed by MetaXpress (Molecular Devices) software using the Cell scoring module to obtain the average γ-H2AX fluorescent intensity and using the Granularity module to obtain the γ-H2AX or 53BP1 foci per cell. The primary antibodies involved were anti-γ-H2AX (Ser139) (1:500, CST (Danvers, USA), #9718) and anti-53BP1 (1:250, Abcam (Waltham, USA), #ab175933).

### Western blotting

Cellular proteins were extracted using RIPA lysis buffer (Beyotime) pre-cooled at 4 °C containing 1% PMSF (Beyotime), 1% protease inhibitor cocktail (MCE, New York, USA), and 1% phosphatase inhibitor cocktail (MCE). This was followed by adding 4 × Laemmli Sample Buffer (Bio-Rad, Hercules, USA) and β-mercaptoethanol. After that, incubate for 10 min at 100 °C to denature the proteins.

The gels were prepared according to the instructions of CFAS Any KD PAGE Protein Electrophoresis Gel Preparation Kit (ZHONGHUIHECAI, Xi’an, China). After being separated with a vertical polyacrylamide gel electrophoresis system (Bio-Rad), the proteins were transferred to a 0.45 μm PVDF membrane (Millipore, Bedford, USA). Subsequently, blocking was performed with 5% non-fat milk at room temperature for 1 h. The bands were incubated with the primary antibody at 4 °C overnight and then incubated with horseradish peroxidase-conjugated secondary antibody at room temperature for 1 h. Secondary antibodies were detected using an ECL chemiluminescence kit (Invitrogen) and visualized by an Imaging System (Bio-Rad). The relative protein content was analyzed with Image Lab software (Bio-Rad). Glyceraldehyde-3-phosphate dehydrogenase (GAPDH) is used as the loading control.

The involved primary antibodies were anti-γ-H2AX (Ser139) (1:1000, CST, #9718), anti-BAX (1:1000, Proteintech (Wuhan, China), #50599-2-Ig), anti-Caspase 3 (1:1000, CST, #9662), anti-HO-1 (1:1000, Proteintech, #10701-1-AP), anti-topoisomerase II (1:1000, abcam, #ab109524), and anti-GAPDH (1:1000, Beyotime, #AF0006).

### H9c2-EGFP-53BP1 cell sample preparation, image acquisition, and image analysis

Doxorubicin hydrochloride (MCE), reference compound dexrazoxane (Selleck, Houston, USA), reference compound resveratrol (SIGMA-ALDRICH, Saint Louis, China), and isoliquiritigenin (Yuanye, Shanghai, China) which all have a purity of ≥ 96% were purchased and prepared as a stock solution using DMSO (SIGMA-ALDRICH). Prior to use, the stock solution was diluted to the expected concentration with DMEM complete medium. H9c2-EGFP-53BP1 cells were seeded in black 96-well cell culture plates (Greiner (Monroe, USA), #655090), and treated with or without the indicated concentrations of DOX, and with or without the indicated concentrations of compounds for specific durations before fixation.

Cells were fixed with 4% paraformaldehyde solution for 10 min, then washed twice with PBS. Next, nuclei were stained with 1 μg/mL Hoechst 33,342 for 10 min and then washed three times with PBS. Finally, add PBS to each well to avoid dryness.

The images of DAPI channel and FITC channel were acquired through a 40× objective at room temperature. The imaging devices are as follows. In order to observe the fluorescence distribution of H9c2-EGFP-53BP1 cell lines (Fig. 2B) and to select appropriate treatment duration of DOX (Fig. 2C), Leica DMi3000 B manual microscope was used to carefully focus and obtain images manually one by one. For other H9c2-EGFP-53BP1 images, including images corresponding to Fig. 2E, Fig. 3A–B, Fig. 5A, Additional file 3: Fig. S3 and Additional file 4: Fig. S4, were all acquired using the ImageXpress PICO (Molecular Devices) or ImageXpress Micro Confocal (Molecular Devices) high-content imaging systems. It should be pointed out that different imaging devices were used in Fig. 2C and E, which may be the reason why there is a statistically significant difference of foci per cell between the control group and 0.5 µM DOX 1 h group in Fig. 2D, while there is no statistically significant difference between the control group and 0.5 µM DOX 1 h group in Additional file 5: Fig. S5A.

For the threshold-based traditional method, the images were analyzed using the Granularity module in MetaXpress software to obtain the number of foci in the nucleus. For the deep learning-based method, images were analyzed using the FociNet in Python to obtain the percentage of foci-positive cells. The percentage of foci-positive cells = the number of foci-positive cells/(the number of foci-positive cells + the number of foci-negative cells) × 100%.

### High-content screening

H9c2-EGFP-53BP1 cells were seeded in black 96-well cell culture plates at 3000 cells/well. A normal control group and a DOX group were set up on each plate. The compounds to be tested were 315 compounds from the self-made compound library of the lab. Twenty-four hours after seeding, the cells were incubated with 0.5 μM DOX and 50 μM compounds for 1 h. Then, the cells were fixed, stained with Hoechst, and placed into the ImageXpress Micro Confocal imaging system to obtain images of the DAPI channel and the FITC channel through a 40× objective.

The high-content screening images were analyzed using both the threshold-based traditional method and the deep learning-based method, FociNet. In the threshold-based method, MetaXpress was used to segment the cells and recognize the foci. The number of foci per cell in each well was obtained, and the normalized number of foci was calculated according to the following formula [46]: Robust Percent of Samples (RPS) = S_i_ ÷ median(S_all_) × 100. For this calculation, RPS = value of the compound ÷ median value of all compounds in the same plate × 100. In FociNet analysis, the percentage of foci-positive cells in each well was obtained and then normalized by the same RPS formula. For both image analysis methods, the mean value of the three RPSs obtained from the three experiments was used to rank the compounds, and the 12 compounds with the lowest mean RPS were selected for subsequent experiments.

### Measurement of cellular ATP levels

H9c2 cells were seeded in white opaque 96-well cell culture plates. Cellular ATP levels were measured using the CellTiter-Glo kit (Promega, Madison, USA). The CellTiter-Glo solution was prepared according to the instructions and was added to the wells of the cell plate, shaken, and incubated at 37 °C for 10 min. Finally, the chemiluminescence intensity values were measured by a microplate reader (TECAN, Männedorf, Switzerland). The relative ATP level was calculated by dividing the value of each group by the value of the normal control group.

### Apoptosis/necrosis assay

H9c2 cells were seeded in black 96-well cell culture plates. Twenty-four hours after seeding, cells were incubated with DOX and ISL for 24 h. Then, apoptosis/necrosis assays were performed using the Annexin V-FITC kit (Beyotime). A mixture was prepared by mixing Annexin V-FITC conjugate and Annexin V-FITC solution at a ratio of 39:1, and the mixture was then supplemented with 10 μg/mL Hoechst 33,342 solution to get the working solution. For staining, the plates were centrifuged at 1000 RCF for 5 min, and then the cells were washed once with PBS. After aspirating the PBS, staining working solution was added to each well in a light-protected environment and incubated at room temperature for 15 min, followed by immediate image acquisition on the ImageXpress Micro Confocal imaging system using a 20× objective, with DAPI channel and FITC channel selected. The percentage of Annexin V-FITC-positive (apoptotic/necrotic) cells was analyzed using the Cell Scoring module in MetaXpress software.

### Detection of cellular peroxynitrite

The B545b probe used for the detection of peroxynitrite (ONOO^−^) was a gift from Professor Xin Li [47]. The B545b was prepared as a 5 mM stock solution in DMSO, stored at − 20 °C. Before use, prepare the working solution by diluting the B545b to 5 μM with DMEM medium and adding Hoechst to a final concentration of 10 μg/mL. H9c2 cells were seeded in black 96-well cell culture plates with 3000 cells per well. Twenty-four hours after seeding, cells were incubated with 0.5 μM DOX and specific concentration of ISL for 24 h. For staining, cells were washed three times with PBS and then stained with B545b working solution at 37 °C for 20 min. The ImageXpress Micro Confocal Imaging System chamber was pre-heated to 37 °C, and DAPI-channel and TRITC-channel images were acquired through the 60× objective. The TRITC-channel B545b fluorescent signal was analyzed using the Cell Scoring module in the MetaXpress software.

### Image adjustment and display

For EGFP-53BP1-H9c2 images, in order to show the distribution of EGFP-53BP1 protein more clearly, we performed different brightness adjustment for different images. Nevertheless, for the experiments where the fluorescent intensity is correlated with the concentrations of interested substances (i.e., γ-H2AX immunofluorescence, 53BP1 immunofluorescence, apoptotic/necrotic cells labeling with Annexin V-FITC, peroxynitrite labeling with B545b), quantitative analysis was performed on unadjusted 16-bit raw images, and the corresponding representative images have undergone the same brightness adjustment.

### Molecular docking

The docking software was the Glide module of Schrödinger 2018 (Schrödinger, LLC, New York, USA). The crystal structure 1ZXM was processed by the Protein Preparation Wizard module of the Schrödinger software. The processing includes completing the missing side chain and loop region structure of the protein, removing all unbonded heteroatoms and water molecules, completing the missing hydrogen atoms, assigning protonated states and partial charges, and finally optimizing the protein structure using the OPLS3e force field until RMSD is optimized to a maximum value of 0.3 Å to reduce atomic space collisions. Next, a box of 20 Å × 20 Å × 20 Å centered on the center of mass of the ligand was identified as the docking area using the Receptor Grid Generation module, and the grid file for docking was generated.

The 3D structure of the small molecule, ISL, was preprocessed with the Ligprep module, and the protonated state was generated at pH = 7.0 ± 2.0. The prepared small molecule was then docked to the pocket using the Glide program in standard scoring mode (standard precision, SP).

### MTT assay for cell viability

Thiazolyl Blue Tetrazolium Bromide (MTT, Sigma-Aldrich) solution was prepared as 5 mg/mL in PBS, and the solution was filtered with a 0.22 μm filter for sterilization and diluted to 0.5 mg/mL with DMEM medium right before use. After three times washing with DMEM medium, cells were incubated with 0.5 mg/mL MTT in the CO_2_ incubator for 4 h. Then, replace the MTT solution in the wells with 100 μL DMSO, shake the plate for 10 min at 37 °C, and measure the absorbance at 570 nm in a microplate reader (TECAN). The absorbance of the treated group was divided by the absorbance of the control group to calculate the relative cell viability.

### Animal experiments

Male C57BL/6 mice aged 6 ~ 8 weeks and weighing 22 ~ 26 g were purchased from Shanghai SILAIKE Laboratory Animal Company. The animal experiments were carried out following the guidance of the Care and Use of Laboratory Animals published by the US National Institutes of Health (NIH Publication No.85–23, revised 1996) and the protocols were approved by the Laboratory Animal Ethics Committee of Zhejiang University (Permit number: ZJU20220096).

The mice were randomly grouped into the normal control group, 15 mg/kg DOX group, DOX + DXZ group, DOX + 10 mg/kg/d ISL group, and DOX + 30 mg/kg/d ISL group. Except the normal control group, all mice were injected with 15 mg/kg DOX intraperitoneally and the day of injection was recorded as Day 1. DXZ was administered intraperitoneally at 60 mg/kg on Day 1 and Day 5. ISL was given via an intragastric administration once daily for 8 days (10 mg/kg/d or 30 mg/kg/d). The doses of ISL, DOX and DXZ were selected according to previously reported studies, respectively [48–50].

### Transmission electron microscopy of myocardial tissue

Myocardial tissue samples of about 1 mm × 1 mm × 4 mm were cut out and immersed in Gluta fix solution (Solabio, Beijing, China), fixed overnight at room temperature, and transferred to 4 °C. The reagents used in the sample preparation were provided by the Electron Microscopy Center of Zhejiang University.

The tissues were rinsed three times with 0.1 M PBS for 10 min each. They were fixed with 100 μL of 1% osmium acid for 1 h, then rinsed 3 times with water for 10 min each. After that, fix/stain them with 100 μL 2% uranyl acetate for 30 min. Then, the samples were dehydrated by soaking in a gradient of 50% ethanol, 70% ethanol, 90% ethanol, and 100% ethanol each for 15 min, and then soaked in 100% acetone twice for 15 min each time. The samples were then permeabilized overnight at room temperature with a mixture of 3:1 embedding agent:100% acetone. Finally, embedding, sectioning, and image acquisition were performed by the Electron Microscopy Center of Zhejiang University, where images of myofilaments and mitochondria of myocardial tissue were acquired using 120 kV cryo-transmission electron microscopy.

### Echocardiographic evaluation of cardiac function

M-mode echocardiograms were recorded on Day 9. The measurements were performed by experienced operators who were blinded to the study group assignment. Echocardiographic images of the left ventricle of the mice were collected in the anterior thoracic region after the removal of hair. The data were analyzed and calculated in Vevo2100 (Visualsonics, Toronto, Canada) to obtain the ejection fractions (EF) and fractional shortening (FS) of the left ventricle.

### Statistical analysis

GraphPad Prism (San Diego, USA) was used for statistical analysis of the experimental data. Kruskal–Wallis test was performed for Fig. 1B and Fig. 5C, and Mann–Whitney test was performed for Fig. 4B (c) and Additional file 5: Fig. S5C (middle). For other data, if not specifically stated, two-tailed unpaired t test was employed to make comparisons between two groups, and One-Way ANOVA with Dunnett’s multiple comparisons test was performed for comparisons between multiple groups.

### Supplementary Information

**Additional file 1**. **Fig. S1** Representative immunofluorescence images of DOX-treated H9c2 and the number of cells quantified. (A) Representative images of H9c2 after incubation with different concentrations of DOX for 1 h or 24 h, γ-H2AX labeled by immunofluorescence. (B) The number of cells that were captured and quantified in γ-H2AX immunofluorescence, corresponding to Fig. 1E. (C) The number of cells that were captured and quantified in 53BP1 immunofluorescence, corresponding to Fig. 1G. For (B) and (C), data are plotted as mean ± SD, n = 3, *P < 0.05, **P < 0.01 when compared with the vehicle (dimethyl sulfoxide, DMSO)-treated control group.

**Additional file 2**. **Fig. S2** The quantified results of western blotting. Data are plotted as mean ± SD, n = 3, ^#^*P* < 0.05, ^##^*P* < 0.01 when compared with the control group; *P < 0.05, **P < 0.01 when compared with the DOX group.

**Additional file 3**. **Fig. S3** The comparison of threshold-based method and FociNet in detecting nuclei boundaries when there are cells with different brightness of nuclear staining.

**Additional file 4**. **Fig. S4** The comparison of threshold-based method and FociNet in detecting foci when there are cells with different EGFP-53BP1 brightness.

**Additional file 5**. **Fig. S5** The analysis result of the threshold-based traditional method on the images of cells treated with reference compounds and the images of the high-content screening. (A) The effect of reference compounds on foci formation was analyzed by the threshold-based traditional method. Data are plotted as mean ± SD, n = 3. Comparisons between the DOX group and every other group were performed by ANOVA. The *ns* indicates no significant difference; ^*^P < 0.05. (B) Scatter plots of the primary screening. The mean values of foci per cell calculated by the threshold-based method are presented as RPS, and the blue data points represent hit compounds. (C) Cumulative distribution of the primary screening data (left). Scatter plot (middle) and frequency distributions (right) of data from DMSO-treated control wells *vs.* hit compounds-treated wells. For (C, middle), data are plotted as mean ± SD, n = 36 ~ 54, ^**^P < 0.01 by Mann–Whitney test.

**Additional file 6**. **Fig. S6** In the secondary screening, 12 of the 17 primary hit compounds significantly reduced the foci at 50 μM. Data are plotted as mean ± SD, n = 3, ^##^*P* < 0.01 when compared with the control group; *P < 0.05, **P < 0.01 when compared with the DOX group.

**Additional file 7**. **Fig. S7** The IC_50_ of ISL. (A) The inhibition effect of ISL on DOX-induced foci in EGFP-53BP1-H9c2 and the calculated IC_50_. (B) The inhibition effect of ISL on DOX-induced apoptosis/necrosis in H9c2 cardiomyocytes and the calculated IC_50_. Data are plotted as mean ± SD, n = 3.

## Data Availability

The data and materials in the study are available from the corresponding author on reasonable request.

## Abbreviations

53BP1: P53-binding protein 1
AIC: Anthracycline-induced cardiotoxicity
BAX: Bcl-2 associated X-protein
DOX: Doxorubicin
DSB: Double-strand break
DXZ: Dexrazoxane
EGFP: Enhanced green fluorescent protein
GAPDH: Glyceraldehyde-3-phosphate dehydrogenase
HO-1: Heme oxygenase-1
ISL: Isoliquiritigenin
RES: Resveratrol
ROS: Reactive oxygen species
topo II: Topoisomerase II
γ-H2AX: Phosphorylated histone H2AX
