# Supplementary figures and images for: Deep learning-assisted high-content screening identifies isoliquiritigenin as an inhibitor of DNA double-strand breaks for preventing doxorubicin-induced cardiotoxicity
Source: Biol Direct. 2023 Oct 9;18:63. doi: 10.1186/s13062-023-00412-7 (PMC10561451; doi:10.1186/s13062-023-00412-7)

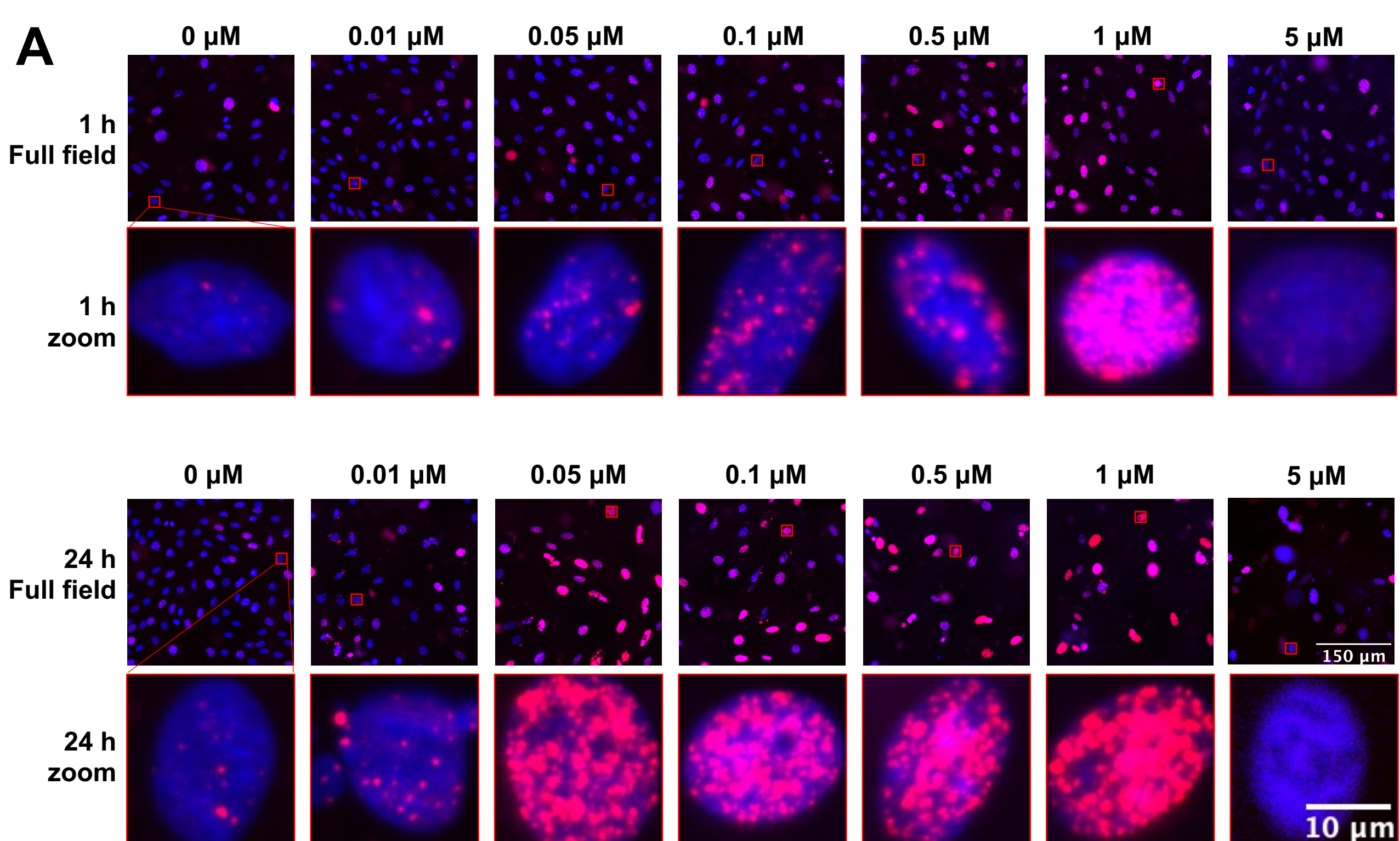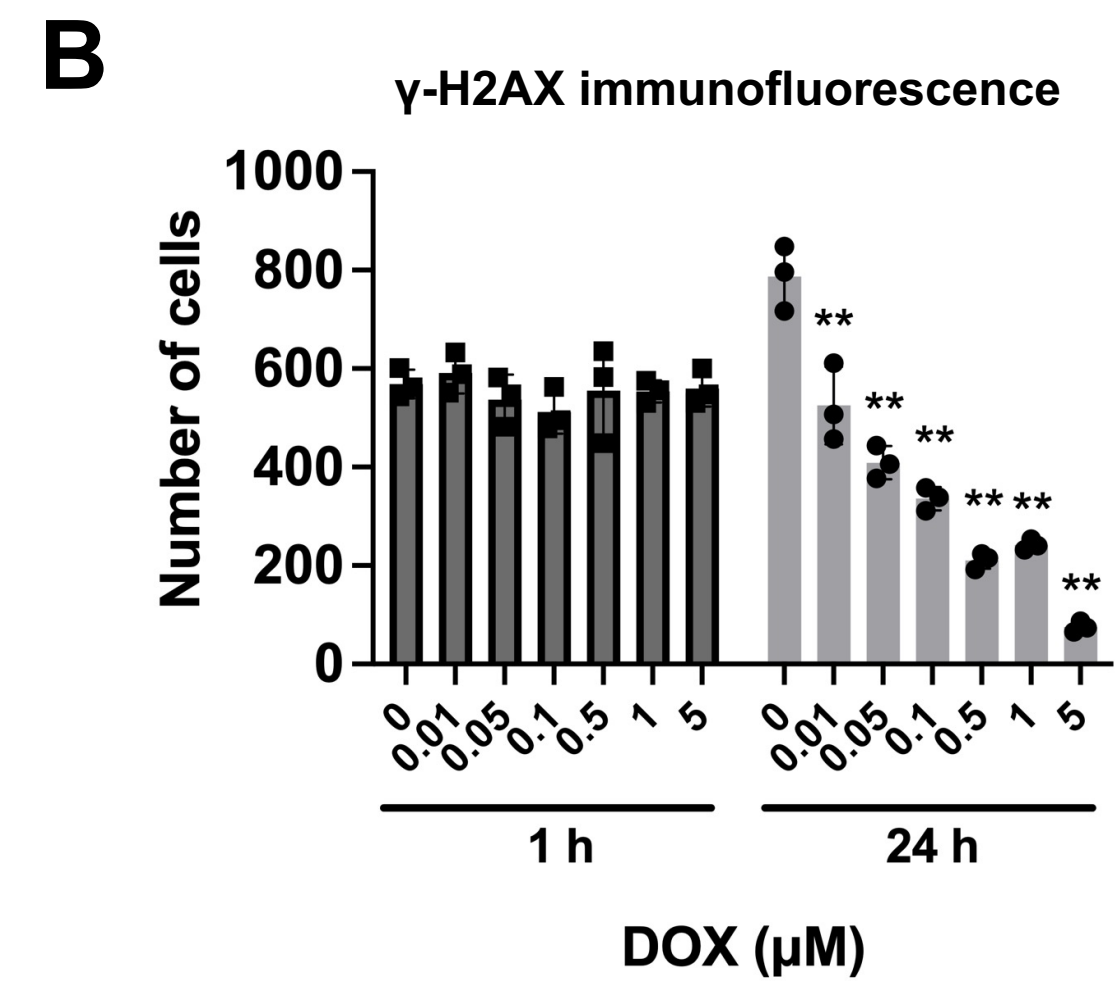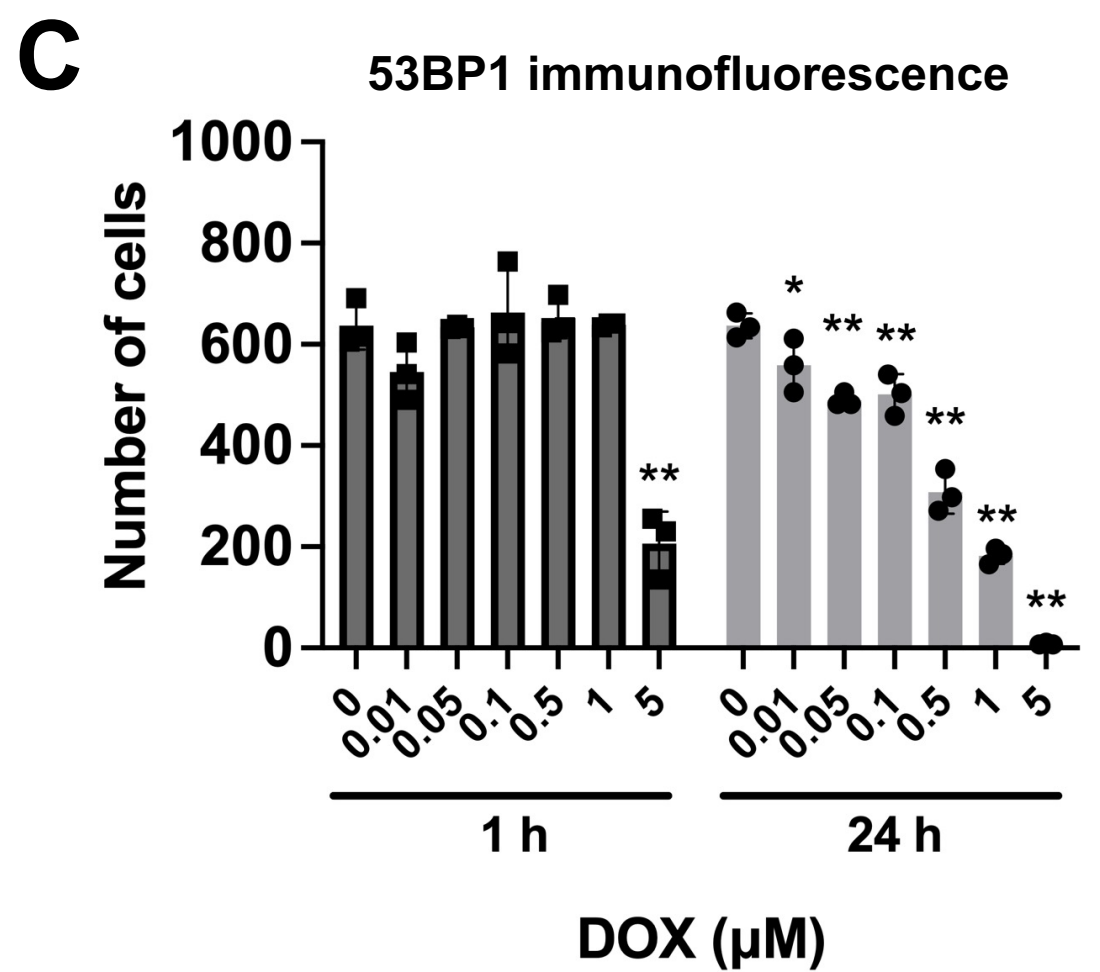

Supplement: Supplementary file 1 — Additional file 1. Fig. S1 Representative immunofluorescence images of DOX-treated H9c2 and the number of cells quantified. (A) Representative images of H9c2 after incubation with different concentrations of DOX for 1 h or 24 h, γ-H2AX labeled by immunofluorescence. (B) The number of cells that were captured and quantified in γ-H2AX immunofluorescence, corresponding to Fig. 1E. (C) The number of cells that were captured and quantified in 53BP1 immunofluorescence, corresponding to Fig. 1G. For (B) and (C), data are plotted as mean ± SD, n = 3, *P < 0.05, **P < 0.01 when compared with the vehicle (dimethyl sulfoxide, DMSO)-treated control group. [file 13062_2023_412_MOESM1_ESM.pdf]

**A**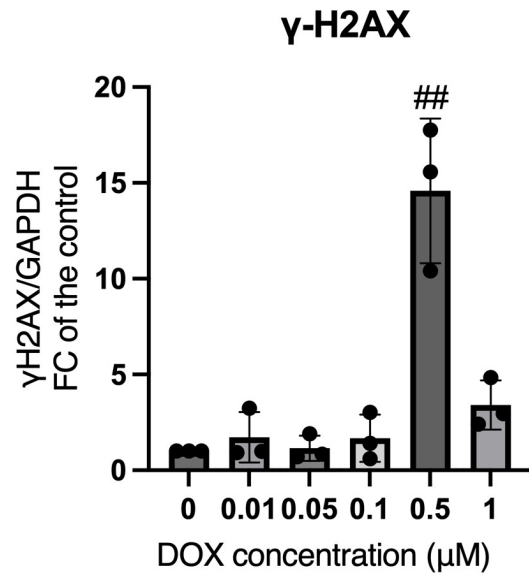**B**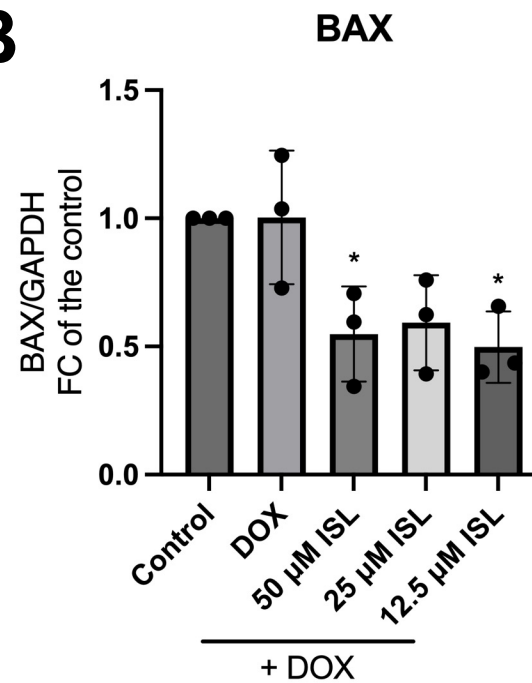**C**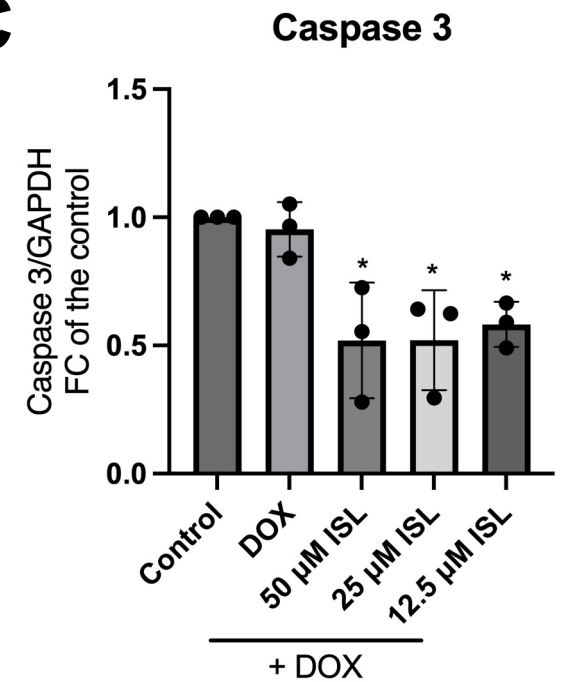**D**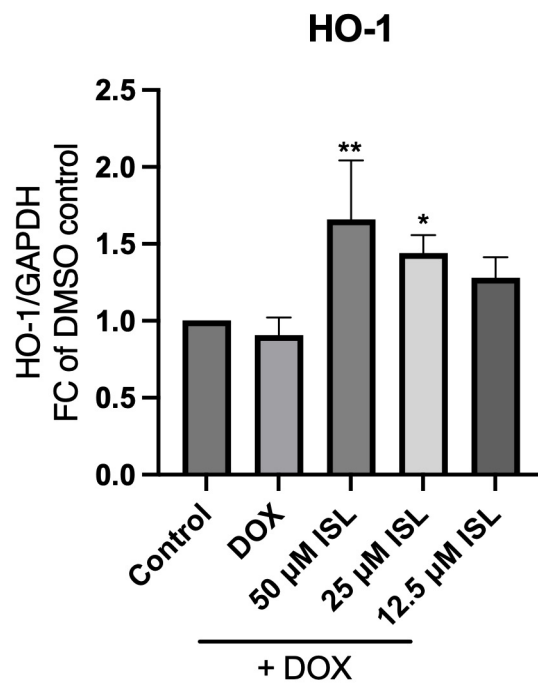**E**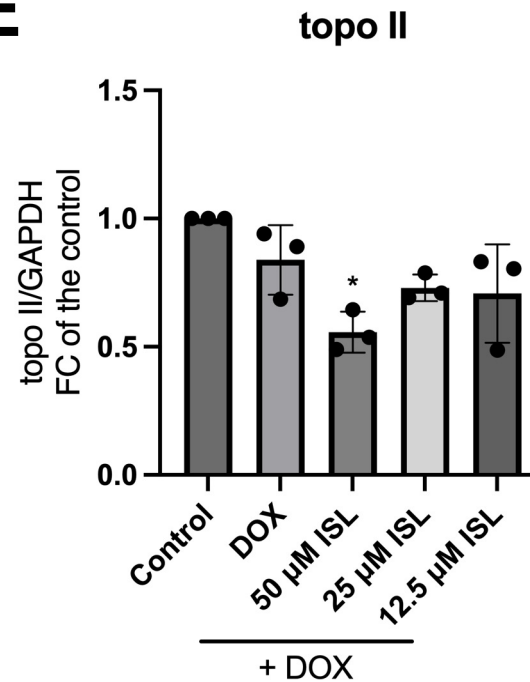

Supplement: Supplementary file 2 — Additional file 2. Fig. S2 The quantified results of western blotting. Data are plotted as mean ± SD, n = 3, #P < 0.05, ##P < 0.01 when compared with the control group; *P < 0.05, **P < 0.01 when compared with the DOX group. [file 13062_2023_412_MOESM2_ESM.pdf]

**A**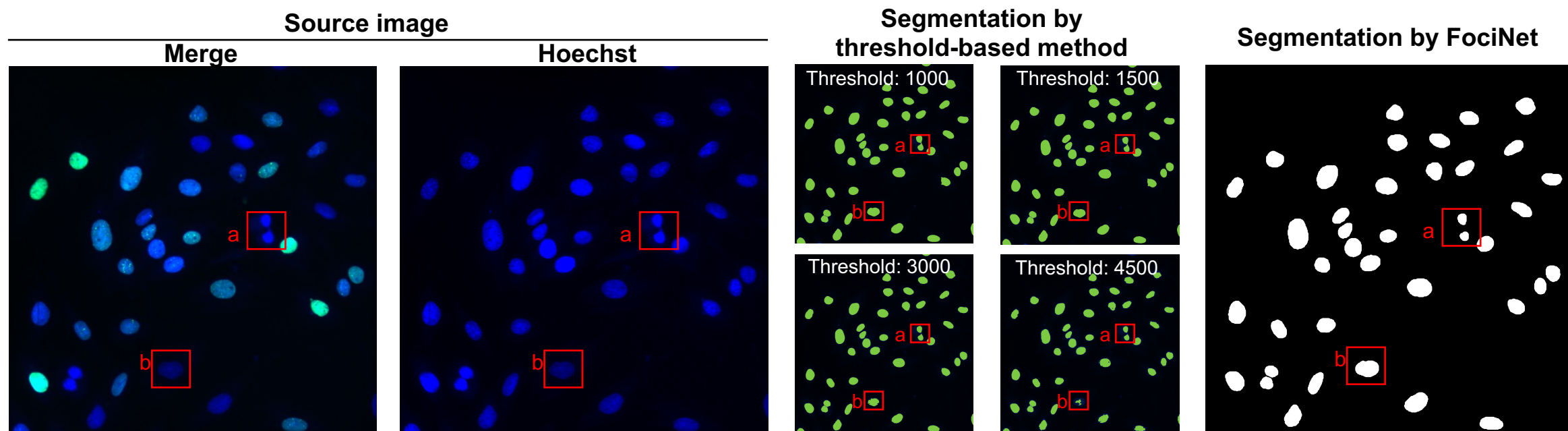**B**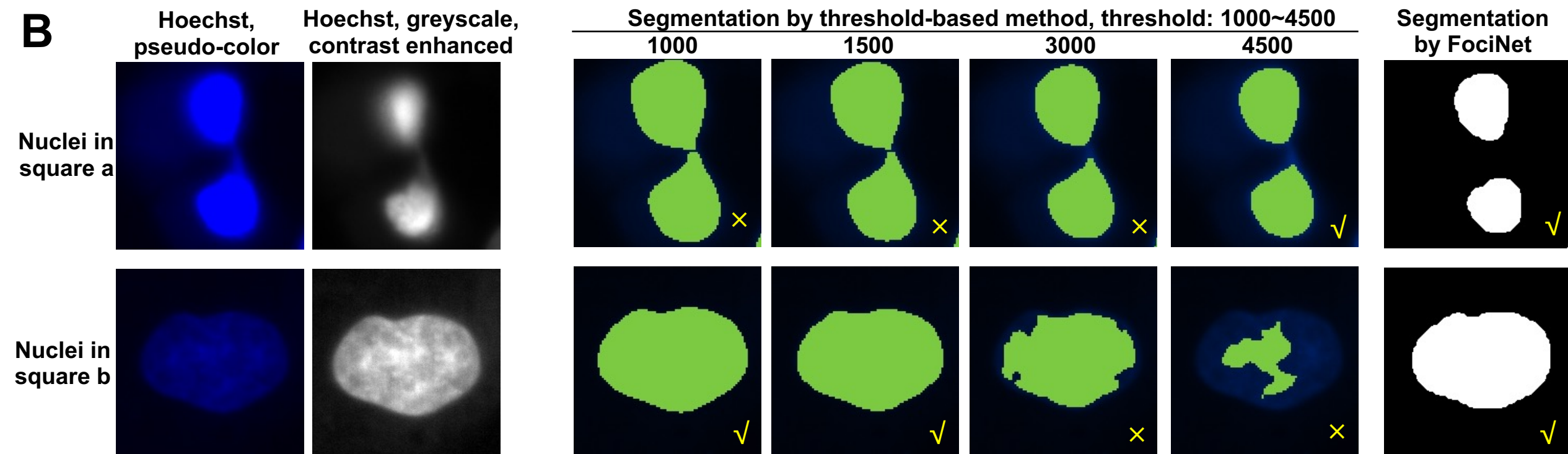

Supplement: Supplementary file 3 — Additional file 3. Fig. S3 The comparison of threshold-based method and FociNet in detecting nuclei boundaries when there are cells with different brightness of nuclear staining. [file 13062_2023_412_MOESM3_ESM.pdf]

**A**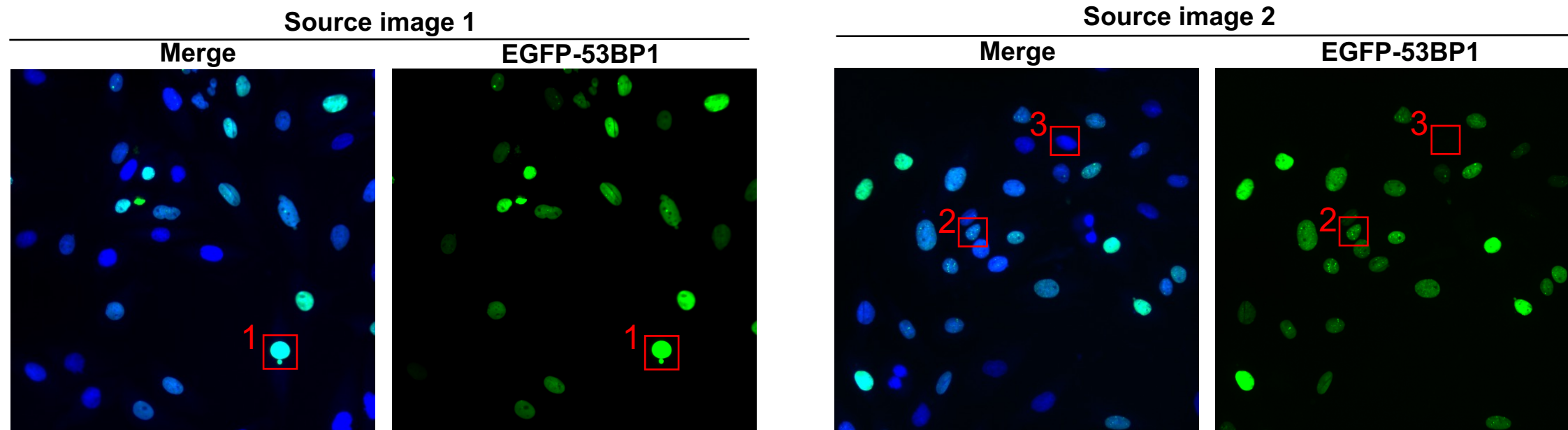**B**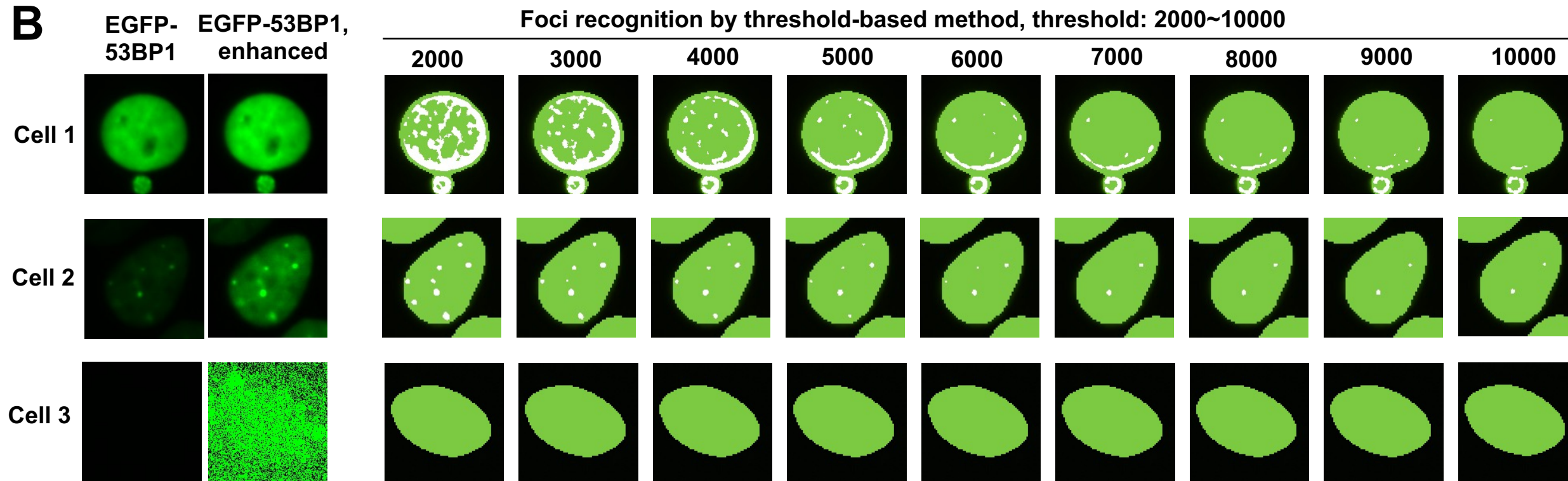

Supplement: Supplementary file 4 — Additional file 4. Fig. S4 The comparison of threshold-based method and FociNet in detecting foci when there are cells with different EGFP-53BP1 brightness. [file 13062_2023_412_MOESM4_ESM.pdf]

**A**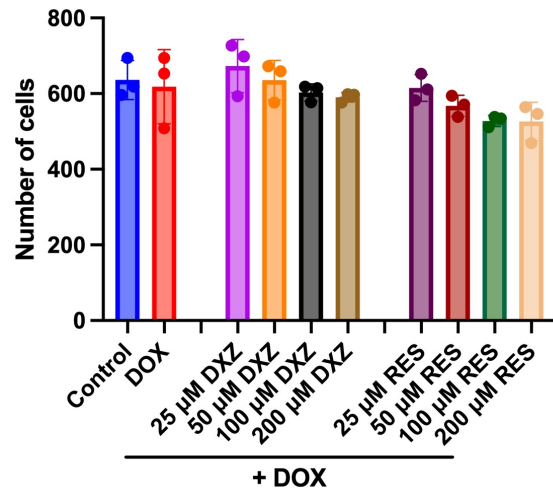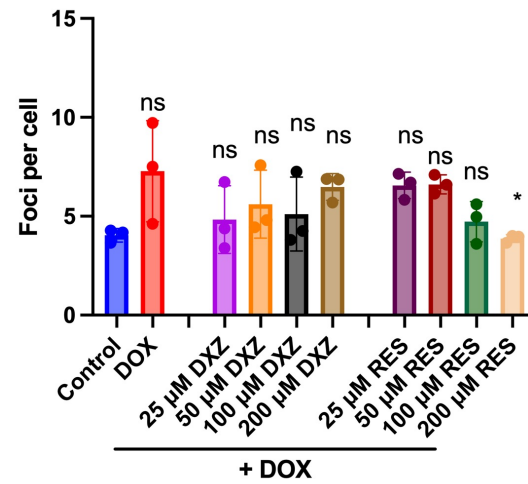**B**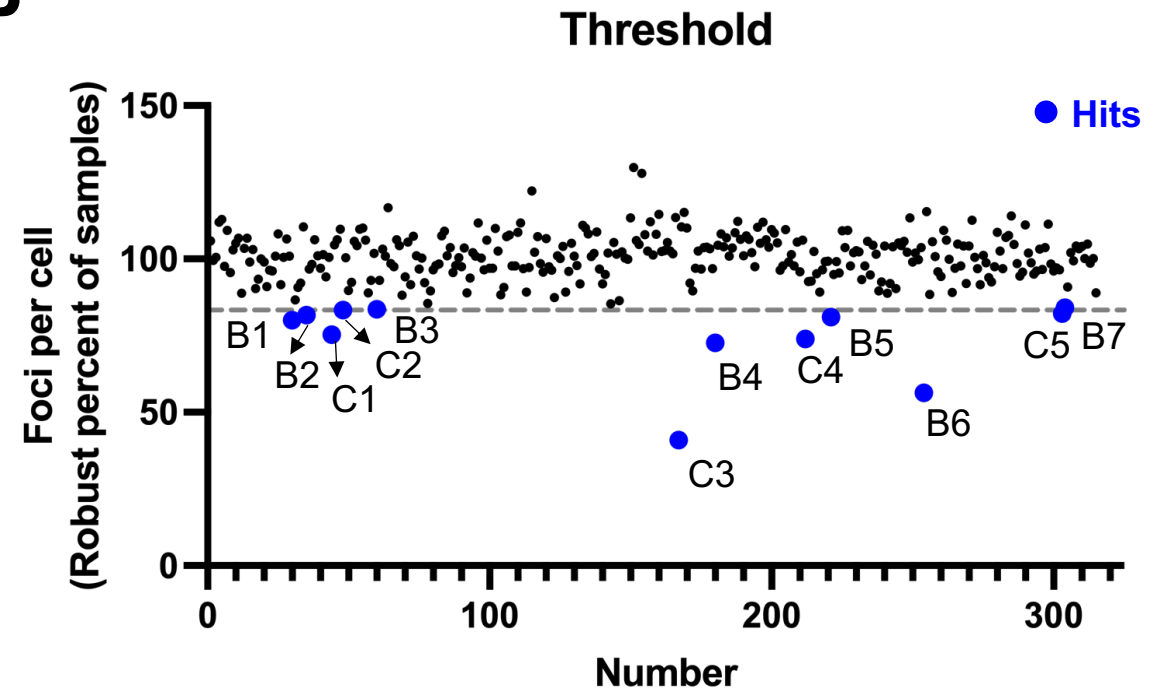**C**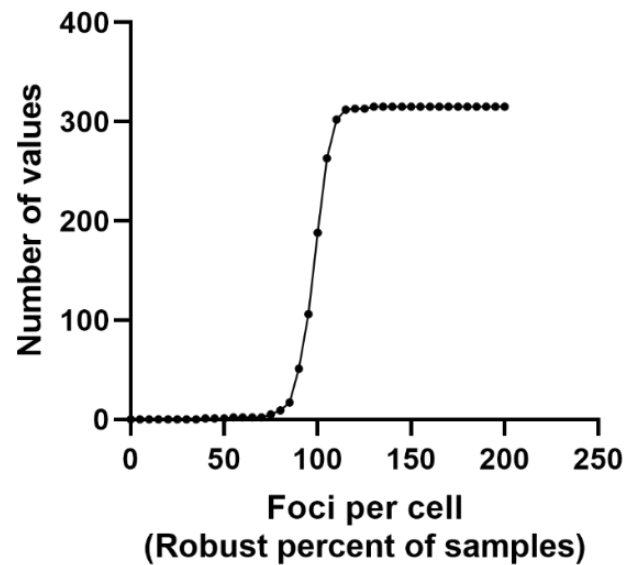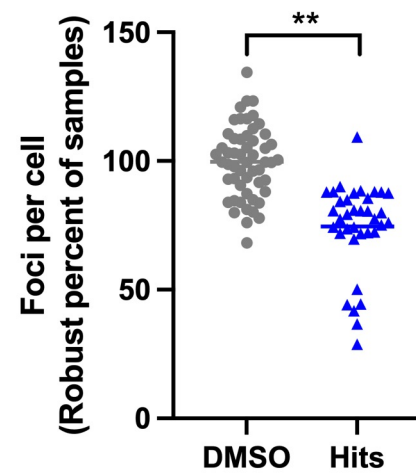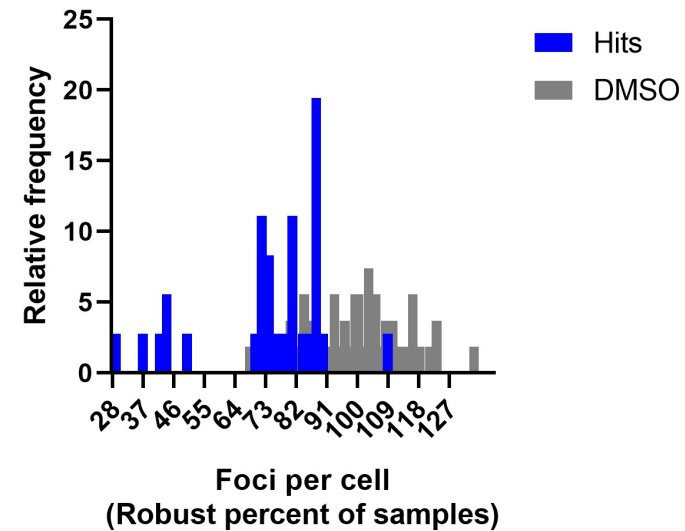

Supplement: Supplementary file 5 — Additional file 5. Fig. S5 The analysis result of the threshold-based traditional method on the images of cells treated with reference compounds and the images of the high-content screening. (A) The effect of reference compounds on foci formation was analyzed by the threshold-based traditional method. Data are plotted as mean ± SD, n = 3. Comparisons between the DOX group and every other group were performed by ANOVA. The ns indicates no significant difference; *P < 0.05. (B) Scatter plots of the primary screening. The mean values of foci per cell calculated by the threshold-based method are presented as RPS, and the blue data points represent hit compounds. (C) Cumulative distribution of the primary screening data (left). Scatter plot (middle) and frequency distributions (right) of data from DMSO-treated control wells vs. hit compounds-treated wells. For (C, middle), data are plotted as mean ± SD, n = 36 ~ 54, **P < 0.01 by Mann–Whitney test. [file 13062_2023_412_MOESM5_ESM.pdf]

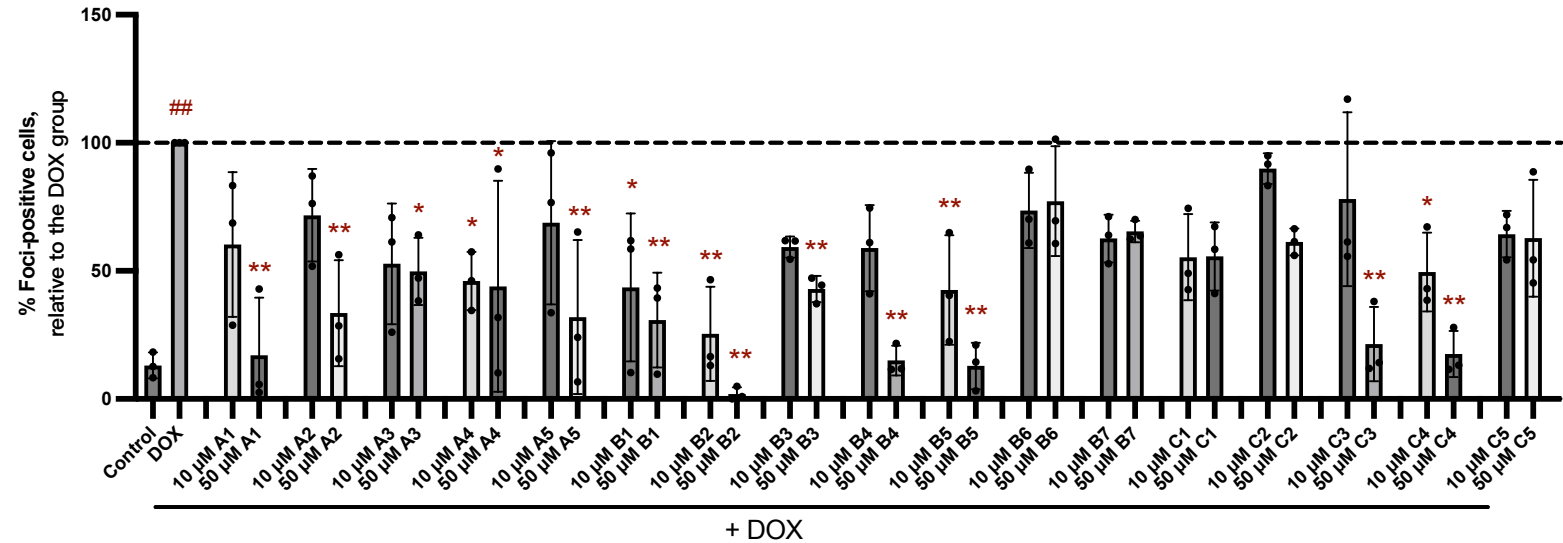

Supplement: Supplementary file 6 — Additional file 6. Fig. S6 In the secondary screening, 12 of the 17 primary hit compounds significantly reduced the foci at 50 μM. Data are plotted as mean ± SD, n = 3, ##P < 0.01 when compared with the control group; *P < 0.05, **P < 0.01 when compared with the DOX group. [file 13062_2023_412_MOESM6_ESM.pdf]

**A**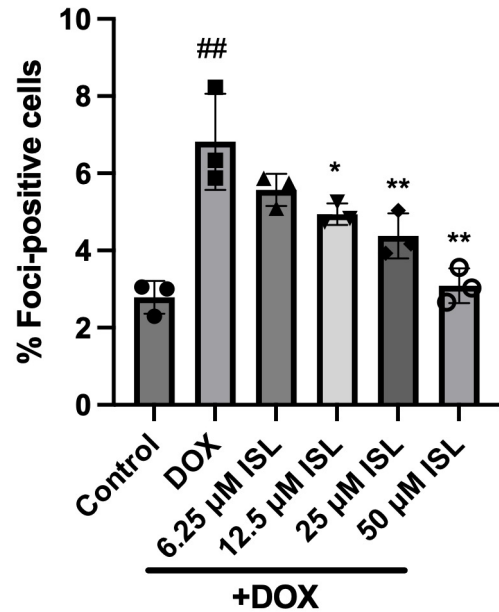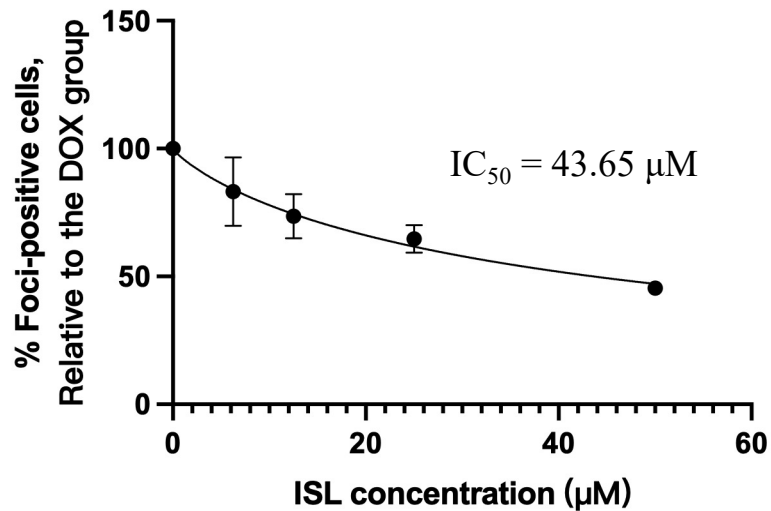**B**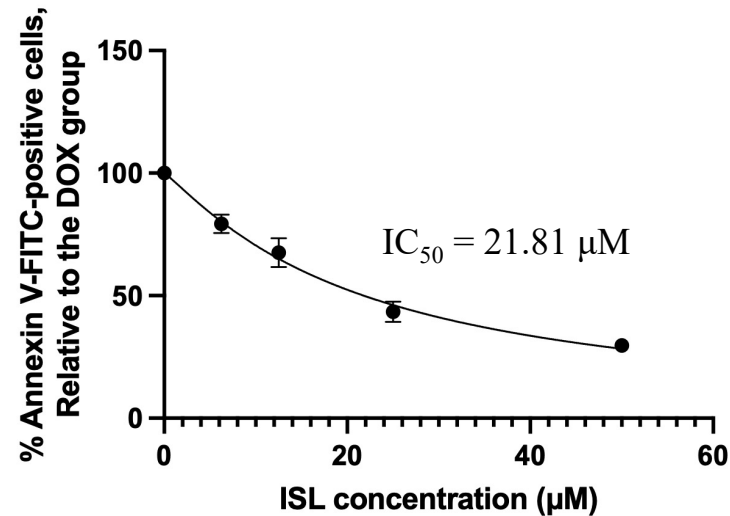

Supplement: Supplementary file 7 — Additional file 7. Fig. S7 The IC50 of ISL. (A) The inhibition effect of ISL on DOX-induced foci in EGFP-53BP1-H9c2 and the calculated IC50. (B) The inhibition effect of ISL on DOX-induced apoptosis/necrosis in H9c2 cardiomyocytes and the calculated IC50. Data are plotted as mean ± SD, n = 3. [file 13062_2023_412_MOESM7_ESM.pdf]
